# Directive vs. Reductive Front-of-Pack Labels: Differences in Italian Consumers’ Responses to the Nutri-Score and the NutrInform Battery

**DOI:** 10.3390/foods14234033

**Published:** 2025-11-25

**Authors:** Nazarena Cela, Federica Quintiero, Cinzia Ferraris, Luisa Torri

**Affiliations:** 1 University of Gastronomic Sciences, 12042 Bra, Italy; n.cela@unisg.it; 2 Laboratory of Food Education and Sport Nutrition, Department of Public Health, Experimental and Forensic Medicine, University of Pavia, 27100 Pavia, Italy; federica.quintiero@unipv.it (F.Q.); cinzia.ferraris@unipv.it (C.F.)

**Keywords:** cognitive abilities, consumer perception, eating disorder, healthy eating, orthorexia nervosa, nutritional label, purchase decision-making, sociodemographic

## Abstract

There is no clear consensus regarding which Front-of-Pack (FoP) label is more effective in promoting healthier food choices. This study explored consumers’ healthiness perception (HP) and willingness to buy (WTB) foods labelled with two different FoP labels: Nutri-Score (NS) and NutrInform Battery (NIB). The role of individual characteristics, such as sociodemographic variables, purchasing behaviors, orthorexia nervosa tendency, and cognitive abilities, in predicting consumers’ responses was also examined. Through an online survey, Italian consumers (*n* = 436; 71% female; average age: 38.9 ± 14.7) evaluated the HP and WTB of yoghurt and fruit jam, with three different nutritional qualities (high, medium, low) and labelled with both NS and NIB. The results showed significant differences between NS and NIB, with effects varying across product categories and nutritional profiles. Age, frequency of nutrition label reading, and role in buying decisions emerged as significant predictors of consumers’ responses, particularly for products with high nutritional quality. Conversely, orthorexia nervosa tendencies and cognitive abilities did not significantly predict differences in HP and WTB between FoP labels. These findings expand the understanding of the complexity involved in selecting an appropriate FoP labelling system and offer valuable insights to effectively guide healthier food choices while accommodating diverse consumers’ profiles.

## 1. Introduction

Balanced nutrition is one of the key aspects for the prevention of many chronic noncommunicable diseases, such as obesity, diabetes, and cardiovascular disease [1]. In recent years, there has been an increasing focus on the importance of providing consumers with clear and understandable information to quickly assess the nutritional value of food products when making a purchase [2,3,4,5,6,7]. To this end, the World Health Organization has proposed Front-of-Pack (FoP) labelling as tool to help consumers to make healthier food choices [8]. Different formats of FoP labels adopted in different countries were described and reviewed by Storcksdieck et al. [9]. There are non-colored nutrient-specific labels, such as NutrInform Battery used in Italy or the Reference Intake label, which inform consumers about nutrients such as fat, sugar, salt, saturated fat, and calorie content. Conversely, colored nutrient-specific labels, such as Multiple Traffic Light adopted in the UK, combined a three-color code with information related to nutrients. Other formats provide overall summary scoring by using a graded scale, such as that for the Nutri-Score used in France, Luxembourg, Austria, France, the Netherlands, Switzerland, Germany, Belgium, Spain, Portugal, Hungary, Slovakia, etc., or using an endorsement logo, such as the Nordic Keyhole used in Norway, Sweden, and Denmark, or the Healthier Choices adopted in Singapore. There are also warning logos, such as those used in Chile, Uruguay, and Peru, affixed on food products when they exceed thresholds for unhealthy nutrients [10,11]. Moreover, it is possible to group FoP labelling schemes in three types according to the “degree of directiveness”, as proposed by the European taxonomy [12]: (i) non-interpretative/reductive systems, which provide only nutritional information; (ii) interpretative/directive labels, which provide summary information on the overall healthiness or unhealthiness of the product but without giving any specific nutrient information; (iii) semidirective systems, which provide nutritional information complemented by color according to the nutrient levels or by an overall level of healthiness [13,14]. However, there is not a harmonized FoP label policy throughout countries [14]. Indeed, among 44 countries which have introduced FoP labelling schemes as supplementary nutrition information, only 16 have adopted them as mandatory, while the others have implemented them only voluntarily [13]. The European Commission initially announced its intention to propose a harmonized and mandatory FoP label for all member states by the end of 2022; however, the proposal was postponed due to challenges in selecting the appropriate FoP labelling system [15].

Despite the widespread use of FoP labels, some questions emerge regarding their effectiveness and how they are perceived by consumers [16]. To this end, the existing literature has already explored the effectiveness of FoP labels in improving perceptions of food healthfulness and investigated consumers’ preference for different types of labelling [17,18,19]. Among the most debated FoP labelling schemes, there is the Nutri-Score, initially launched in France in 2015 and subsequently adopted by several European countries [20]. The Nutri-Score is a directive FoP labelling format, using a color-coding system (ranging from dark green to red) and a letter (ranging from A to E), thus indicating the overall healthiness level of the food product. This synthetic method aims to make the nutritional quality of a food product immediately visible to the consumer [21]. In Italy, it has been developed the NutrInform Battery system that, as opposed to Nutri-Score, is based on a different approach [22]. Indeed, NutrInform Battery is a reductive/non-interpretative FoP labelling system since it uses the symbol of a cell phone battery to measure the daily intake of five components—calories, fat, saturated fat, sugar, and salt. These batteries display the quantity of each element present in a portion of the food under consideration as well as its contribution to the daily requirement [23,24]. Previous studies compared Nutri-Score and NutrInform Battery in order to identify the most useful in promoting healthier choices [22,24,25,26,27,28,29,30,31]. However, these studies have produced conflicting results, thus revealing that there is no clear consensus regarding which FoP label is more effective. These discrepancies suggested that FoP label performance is not universal, but depends on multiple interacting factors. Indeed, differences in FoP label format (reductive vs. directive), product type, and individual characteristics may have contributed to the observed variability in previous outcomes. Despite these considerations, the existing evidence mainly focused on consumers’ subjective perceptions or objective understanding of FoP labels, without jointly examining whether cognitive evaluations translate into behavioral intentions such as willingness to buy. Moreover, to our knowledge, no previous studies have simultaneously examined how individual factors, such as sociodemographic characteristics, purchasing behavior, eating attitudes, or cognitive abilities, may moderate consumers’ responses to different FoP labelling formats.

To this aim, this study adopted a multidimensional experimental approach aimed at investigating how Nutri-Score and NutrInform Battery affect consumers’ healthiness perception and willingness to buy across food products with different nutritional qualities. By additionally considering the role of individual characteristics, such as sociodemographic variables, purchasing behaviors, orthorexia nervosa tendency, and cognitive abilities, in shaping consumers’ responses, this study was designed to identify potential consumer-related determinants of FoP label effectiveness in order to advance current understanding of the boundary conditions under which directive and reductive FoP labels influence consumer decision-making.

## 2. Theoretical Framework and Research Hypotheses

### 2.1. FoP Labelling Systems

Although FoP labelling systems vary globally and each country may adopt different formats according to national policies [9], the present study focused on Nutri-Score and NutrInform Battery because they represent the two competing approaches currently at the center of the Italian policy debate [27]. Nutri-Score (NS) is currently the most widely adopted FoP label across several European countries [20]. However, its introduction in Italy has been met with debate, with critics arguing that it may oversimplify nutritional information and penalize traditional Italian food products [32,33]. As an alternative, the NutrInform Battery (NIB) was recently developed and proposed in Italy [34]. Several research studies [22,24,26,28] reported that the nutrient-specific and informative format of NIB was better understood by consumers and perceived as more trustworthy, informative, and useful than the interpretive, color-coded NS format. In particular, Castellini et al. [26] found that NIB was more effective in assisting individuals with specific health concerns (e.g., high blood pressure or high cholesterol) in making nutritionally aligned choices, especially among younger and more nutritionally knowledgeable participants. Similarly, Carruba et al. [22] and Mazzù et al. [30] showed that NIB improved consumers’ understanding of nutritional information and could potentially lead to healthier food selections. Additionally, He et al. [28] performed a 20-country comparative assessment to individuate the consumers’ subjective understanding and liking of NS and NIB, highlighting that NIB was more effective than NS in improving consumers’ subjective understanding, both by an aggregated level and by country. Conversely, other studies [27,35] found that the summary, interpretive format of NS was more efficient in helping consumers identify and choose nutritionally favorable products, even among Italian participants who were generally less familiar with this system. Moreover, NS was also perceived as easier to use, leading to higher purchase intentions for healthier options compared to NIB. Therefore, based on the results from previous research, the following research hypothesis was considered in this study:

**H1.**  *There is a significant difference in consumers’ healthiness perception and willingness to buy food products labelled with Nutri-Score and with NutrInform Battery.*

### 2.2. Sociodemographic Characteristics and Purchasing Behavior

Consumers’ sociodemographic characteristics, lifestyle, and dietary factors influenced preference for different formats of FoP labels [36]. For instance, men, older adults, and individuals with poor nutritional knowledge emerged to prefer the simple FoP labelling format [37]. Moreover, individuals who less frequently read nutritional facts on product packages emerged as less skilled at ranking food products according to their nutritional quality than individuals who read nutritional facts more often [38]. Additionally, Baccelloni et al. [25] observed that FoP label performance varied across countries, suggesting the influence of contextual and cultural factors. Based on these previous findings, in this study the following was hypothesized:

**H2.**  *The effect of NS and NIB on healthiness perception and willingness to buy food products depends on consumers’ sociodemographic characteristics and purchasing behaviors.*

### 2.3. Orthorexia Nervosa

One of the eating disorders that has gained more attention in the literature in the last years is orthorexia nervosa (ON), which is characterized by a pathological obsession with consuming foods deemed healthy [39,40,41]. Individuals with this disorder may interpret nutrition labels differently from other consumers, controlling what they eat in an unusual manner [42]. Indeed, as reported by Penzavecchia et al. [6], displaying calorie labels may negatively affect the eating of individuals with eating disorders, even if, on the other hand, the provision of information may reduce feelings of anxiety when eating. Therefore, the nutritional intervention, such as the use of FoP labelling schemes, could have controversial effects on individuals with eating disorders. On the basis of these assumptions, the following hypothesis was defined in this study:

**H3.**  *Individuals with different levels of orthorexia nervosa will tend to have different healthiness perceptions and a willingness to buy NS- and NIB-labelled food products.*

### 2.4. Cognitive Abilities

Consumers’ ability to correctly interpret the information may vary depending on individual characteristics, such as the level of cognitive need, which is the individual’s propensity to engage in complex cognitive activities [43,44]. A previous study showed that individuals with a high need for cognition considered an easy way to use the directive FoP label Keyhole logo to achieve a healthier diet, resulting in higher purchasing intention for Keyhole-labelled products [45]. In addition, the subjective ability to evaluate information is another key variable, as it reflects consumers’ confidence in their ability to correctly understand and interpret the information provided by food labels [24]. Indeed, in a previous study, it was observed that people process food labels in different way: consumers with a more analytical decision-making style preferred detailed nutritional content, fact-based information; those who rely on intuitive processing preferred the simplified FoP system based on visual cues [46]. Therefore, in this study the following was hypothesized:

**H4.**  *The difference in healthiness perception and willingness to buy between NS- and NIB-labelled products is larger in individuals with a high level of need for cognition and subjective ability to evaluate information.*

## 3. Materials and Methods

### 3.1. Study Design

To test the effect of FoP labels on healthiness perception and willingness to buy food products at different levels of nutritional quality, by also taking into account the level of eating disorder and cognitive abilities of consumers, an online experiment was conducted. In total, each participant evaluated 12 experimental conditions: 2 FoP labels (*Nutri-Score* vs. *NutrInform Battery*) × 3 nutritional qualities (*high*, *medium*, *low*) × 2 product categories (*yoghurt* vs. *fruit jam*). Pictures of commercial products were shown in a randomized order among participants to control for order effects. For each picture representing the product, participants were asked to evaluate the healthiness perception of the product and their willingness to buy. Additionally, participants completed other sections of the survey aimed at collecting information about sociodemographic characteristics, purchasing behaviors, eating disorder tendency, and cognitive abilities.

The study was conducted in accordance with the ethical guidelines of the Declaration of Helsinki and received approval from the Ethics Committee of the University of Gastronomic Sciences in Pollenzo, Italy (Ethics Committee Proceedings n. 2023.04).

### 3.2. Front-of-Pack Labels

For this study, two different FoP labels, based on different approaches, were selected: Nutri-Score and NutrInform Battery. The Nutri-Score values for each product were retrieved from the database available in *Open Food Facts* (https://it.openfoodfacts.org/, accessed on 14 September 2023). The NutrInform Battery labels were generated using the official app available on the website https://www.nutrinformbattery.it/ (accessed on 14 September 2023), by entering the nutritional values reported in the corresponding nutritional fact table of each product, also available in the app.

### 3.3. Products and Nutritional Levels

Two product categories were selected as case studies of this study: yoghurt and fruit jam. Both are commonly consumed as part of a typical Italian breakfast [47] and are widely available in supermarkets. The decision to focus on breakfast products aligned with previous research that also compared the performance and consumers’ preferences for NS and NIB but considered other breakfast products, such as biscuits, breakfast cereals, and added fats (i.e., butter) [27]. For each product category, three products were chosen to represent different nutritional qualities: high, medium, and low. This design allowed not only evaluation of the impact of the FoP label itself but also an investigation into whether the possible effect of labelling varies depending on the baseline nutritional quality of the product. Table 1 shows all 12 experimental conditions evaluated in this study.

### 3.4. Survey Sections

An online survey was developed and distributed by using Qualtrics^®^ software (Provo, UT, USA, 2025). The survey consisted of five main sections in the following order: (1) purchasing behavior; (2) FoP label perception assessment; (3) eating disorder; (4) cognitive abilities, such as Need for Cognition and Subjective Ability to Evaluate Information; and (5) sociodemographic characteristics. The complete questionnaire is reported in the Supplementary Material.

Section 1 examined the participants’ purchasing behavior by asking their role in buying decisions and the frequency of reading nutritional labels, adapted from Graham and Laska [48].

In the Section 2, frequency of consumption of both yoghurt and fruit jam was evaluated by answering the following questions: “How often do you consume yoghurt?” and “How often do you consume fruit jam?” (*never, <1 time/month, 1–3 times/month, 1–2 times/week, 3–4 times week, 5–6 times/week, 1/day, >2 times/day*). Then, participants were presented with pictures of the selected products (yoghurt and fruit jam, both at three different nutritional levels), each displayed with either a Nutri-Score and NutrInform Battery label. To avoid brand-related bias, product logos and brand identifiers were digitally removed from all pictures of the products. Additionally, all pictures were standardized to the same size and resolution to minimize perceptual biases related to picture presentation. For each image, participants rated their healthiness perception (HP) of the product, on a scale ranging from 1 (*unhealthy*) to 7 (*very healthy*), and their willingness to buy (WTB), on a scale ranging from 1 (*would definitely not buy it*) to 7 (*would definitely buy it*), as reported by Miraballes & Gámbaro [49].

In the Section 3, the validated Italian version [50] of the Eating Habits Questionnaire-21 (EHQ-21), developed by Gleaves et al. [51], was used to assess the level of orthorexia nervosa (ON). The 21 items were divided into three subscales (*Knowledge*, *Problems*, *Feelings*).

Section 4 investigated the participants’ motivation and ability to assess the quality of information. In particular, the Need for Cognition (NfC) was assessed through the short version (nine items) of the Need for Cognition Scale, as reported by Vainio [44]. In addition, the Subjective Ability to Evaluate Information (SAEI) was evaluated through three items reported by Vainio [44]. Both the NfC and SAEI scales were back translated into the Italian language. The items of the ON, NfC, and SAEI were scored using a seven-point Likert scale ranging from 1 (*strongly disagree*) to 7 (*strongly agree*). These measures were included to capture differences in cognitive engagement and perceived ability to interpret nutrition information beyond what could be explained by formal education alone.

In the Section 5, participants provided their sociodemographic information: *gender, age, education level, area of residence, diet.*

### 3.5. Participants

Participants were recruited by sharing a link through social media channels (LinkedIn, Facebook, WhatsApp), the official website of the NODES project (https://ecs-nodes.eu/en/7-secondary-agroindustry, accessed on 14 September 2023), emails, academic networks, health-related forums, and flyers with a QR code provided at public events. The survey was accessible for seven months (from 21 September 2023 to 5 May 2024) and open to Italian adult (at least 18 years old) consumers. Participants were asked to provide their informed consent before beginning the survey. Responses were collected anonymously. In total, 840 responses were obtained. Participants who did not give their informed consent (*n* = 10) or did not complete all the survey questions (*n* = 282) were not considered for the statistical analysis. Then, 89 participants who completed the questionnaire in a time shorter than 5 min and longer than 25 min were excluded. In addition, participants who stated that they never consume either yoghurt or fruit jam were also excluded (*n* = 23). The final sample size, after removing the “careless respondents” [52,53] and participants who never consume the product categories evaluated in the FoP label perception assessment section, was composed of 436 participants.

### 3.6. Statistical Analysis

To investigate the differences between NS and NIB in terms of HP and WTB, a series of two-sample paired *t*-tests were performed separately for each product category (yoghurt and fruit jam) and for each nutritional quality level (high, medium, low). Moreover, for each product category and for each FoP label, two-way analysis of variance (ANOVA), selecting as a fixed factor the nutritional quality and as a random factor the subject, followed by Tukey’s post hoc test, was performed to individuate differences between the different nutritional qualities, in terms of HP and WTB. When focusing only on yoghurt, participants who reported never consuming yoghurt (*n* = 25) were excluded from the dataset. Therefore, the final total number of participants when considering only yoghurt was 411. Similarly, when analyzing fruit jam, participants who declared never consuming fruit jams (*n* = 65) were excluded from statistical analysis. Therefore, the final total number of participants when considering only fruit jam was 371.

Descriptive statistics (mean ± standard deviation) were computed for each item of the EHQ-21, NfC, and SAEI questionnaires. Then, internal consistency reliability was evaluated using Cronbach’s α coefficients, with values above 0.6 considered acceptable [54,55]. Significant differences among mean values of the three subscales of the EHQ-21 questionnaire were evaluated through ANOVA followed by Tukey’s post hoc test. For each participant (*n* = 436), the total score of the scales of the NfC, SAEI, and EHQ-21 subscales was calculated as the total sums of the score items. Pearson’s correlation analyses were conducted to explore the relationships among the total scores of the subscales of the EHQ-21 questionnaire, NfC, and SAEI scales.

For each product category (yoghurt and fruit jam) and for each nutritional quality (high, medium, low), within-subject changes in healthiness perception (ΔHP) and willingness to buy (ΔWTB) between NS and NIB were calculated as follows:

(1) ΔHP = HP_NIB_ − HP_NS_

(2) ΔWTB = WTB_NIB_ − WTB_NS_

This implied that, when ΔHP > 0, NIB-labelled products were considered healthier than NS-labelled ones. Similarly, when ΔWTB > 0, the purchase intention of NIB-labelled products was higher than those labelled with NS.

To identify the main predictors of ΔHP and ΔWTB, a series of hierarchical multiple linear regression (HMLR) models was performed separately for each product category and each nutritional quality. Sociodemographic characteristics (*gender, age, educational level*) were entered in Model 1. Model 2 added purchasing behavior variables (*role in buying decisions* and *frequency of reading nutritional labels*). Model 3 incorporated orthorexia nervosa total scores derived from the EHQ-21 scale, and Model 4 added cognitive abilities (*NfC* and *SAEI*). For all tests, statistical significance was set at *p* < 0.05. All statistical analyses were performed using XLSTAT Premium software (Version 2020.3.1, Addinsoft, Paris, France), except for the correlation matrix, which was calculated and visualized using the “corrplot” R package (version 4.1.1), and the HMLR analysis, which was performed by using the software IBM SPSS Statistics (Version 29.0.1.0).

## 4. Results and Discussion

### 4.1. Participants’ Profile

#### 4.1.1. Sociodemographic Characteristics and Purchasing Behaviors

As reported in Table 2, the study sample (*n* = 436) participants comprised a majority of women, accounting for a little more than two thirds of the total, while men represented roughly one quarter of the participants. The age distribution revealed that almost half of the participants were young adults between 18 and 35 years old, while the remaining participants were more evenly distributed among the other age groups with the over 61 years group constituting the smallest segment. Regarding the level of education, most participants had a high educational background, with over half holding a university or postgraduate degree and about one fifth possessing a high school diploma, while only a few reported lower levels of education. Geographically, the sample was largely composed of individuals from northern Italy, with a smaller proportion from southern regions. In terms of dietary habits, the majority identified as omnivorous. Concerning food purchasing decisions, nearly half of the participants were primarily responsible for grocery shopping, while a comparable proportion reported sharing this responsibility with other family members. Only a minimal number were not involved in such decisions. Finally, regarding the frequency of reading nutritional labels, approximately one third stated that they often read them, another quarter did so occasionally, whereas only a few reported always or never consulting labels during food purchases.

#### 4.1.2. Orthorexia Nervosa and Cognitive Abilities

The descriptive statistics and reliability analysis of the questionnaires related to orthorexia nervosa and cognitive abilities, reported in the Supplementary Materials (Tables S1 and S2), demonstrated satisfactory internal consistency. The Cronbach’s α of the EHQ-21 was 0.89. Considering the three subscales of the EHQ-21 questionnaire, the Cronbach’s α coefficients ranged from 0.65 (for subscale *Feelings*) to 0.87 (for subscale *Problems*). The *Knowledge* subscale (Cronbach’s α = 0.82), indicating the knowledge of healthy eating [50,51], showed a high mean score (4.7 ± 0.6), suggesting that participants generally perceived themselves as well-informed and health-conscious in their eating habits. The *Feelings* subscale, indicating the positive feeling of people when they eat healthily [50,51], also showed a moderate mean value (4.3 ± 0.5), statistically lower than the mean value of the *Knowledge* scale (*p* < 0.05), reflecting a positive emotional engagement with healthy eating. Conversely, the *Problems* subscale, intended as social problems associated with healthy eating [50,51], showed the statistically lowest mean value (2.4 ± 0.5) among all the subscales (*p* < 0.05), indicating that participants did not report obsession with healthy eating or significant impairment in social life linked to their diet. The mean score of the total EHQ-21 questionnaire (average of all items) was 3.3 ± 1.2, thus suggesting that the participants of this study were a health-oriented sample, but the interest in eating healthily does not becomes an obsession, which is the main characteristic of orthorexia nervosa [51,56].

For the NfC scale, used to evaluate the tendency to assess the quality of information [44], the internal consistency was high (Cronbach’s α = 0.88) and the mean score (3.7 ± 0.6) showed a low–moderate level of cognitive engagement, suggesting that participants were not characterized by strong cognitive abilities but were generally confident making thoughtful decisions. Similarly, the SAEI scale, used to assess the personal skills in assessing the validity and reliability of food-related information [44], demonstrated high reliability (Cronbach’s α = 0.85) with an overall mean of 4.5 ± 0.2, suggesting that participants perceived themselves as relatively capable of distinguishing between valid and invalid information regarding food healthiness and sustainability.

**Figure 1:**
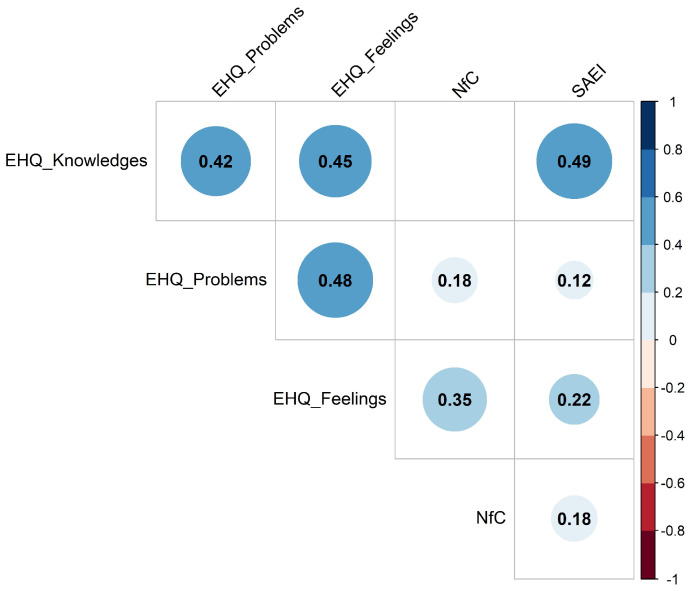
Correlation plot showing the relationships among Eating Habits Questionnaire (EHQ) subscales, Need for Cognition (NfC), and Subjective Ability to Evaluate Information (SAEI) scales (*n* = 436). Blank correlation indicates *p* > 0.05.

The Pearson’s correlations result among the EHQ-21 subscales (*Knowledge*, *Problems*, *Feelings*), NfC, and SAEI scales is reported in Figure 1. Overall, there were significant positive correlations (*p* < 0.05) among the three EHQ subscales, which suggested that individuals reporting more knowledge about healthy eating also reported more diet-related problems and more positive feelings towards healthy eating. The *Knowledge* subscale was moderately and significantly correlated with the *Problems* (r = 0.42) and *Feelings* (r = 0.45) subscales. This result was consistent with previous findings highlighting that excessive focus on weight and food was associated with a reduced emotional state and an increase in the avoidance of social interactions [57], resulting in stricter food choices. The exacerbation of these aspects led to the generation of the key features of orthorexia nervosa [58]. The Problems and Feelings subscales were also moderately and positively correlated (r = 0.48; *p* < 0.05), indicating that even individuals who report issues related to eating healthily still associate it with positive feelings and a sense of control. Indeed, as reported by McCarthy et al. [59], individuals may have hedonistic or emotional motivations for eating, but they exercise self-control to limit their calorie intake in order to maintain a healthy eating routine.

Regarding cognitive abilities, the NfC scale showed weak to moderate significant positive correlation with the *Feelings* subscale (r = 0.35; *p* < 0.05), suggesting that individuals who enjoy and engage in effortful thinking were likely to have more emotional involvement in their healthy food choices. In fact, consumers’ food choices may be influenced by information if consumers are sufficiently motivated to seek out and process that information [44]. Finally, the SAEI scale was also positively correlated with the *Knowledge* subscale (r = 0.49; *p* < 0.05), meaning that individuals perceiving themselves as able in differentiating valid and invalid nutritional information also report higher perceived knowledge of healthy eating, supporting findings from previous literature demonstrating that the ability to evaluate the relevance and quality of information is also important for the comprehension of food labels and hence for making healthier food choices [60]. Therefore, the significant positive correlations between the cognitive aspects (NfC, SAEI) and orthorexia subscales suggested that perceived information-processing ability and needs would modulate how consumers process nutritional information, potentially changing their responses to FoP labelling systems.

### 4.2. Impact of FoP Labels on Healthiness Perception and Willingness to Buy

The results of two-sample *t*-tests on yoghurt are reported in Table 3. For high nutritional quality yoghurt, HP was significantly higher when labelled with NS than with NIB (*p* < 0.05), although no significant difference emerged for WTB. For medium nutritional quality yoghurt, NS obtained higher HP scores than NIB, which also reflected in significantly higher WTB (*p* < 0.05). For low nutritional quality yoghurt, no significant differences between NS and NIB were observed in either HP or WTB (*p* > 0.05). Therefore, for yoghurt of high nutritional quality, NS led to a higher perceived healthiness compared to NIB. This was probably due to the presence of the dark green “A” symbol in NS that was perceived as a positive signal, reinforcing the product healthfulness more effectively than the battery representation in NIB. However, in the low nutritional quality condition, no significant differences were found between the two FoP systems. In this case, both labels presented non-positive signals: the NS showed a yellow “C” code and the NIB batteries highlighted relatively high levels of saturated fats and sugars (20%). Both cues probably guided consumers toward evaluating the product as unhealthy, with mean ratings below the midpoint of the scale. Overall, NS-labelled products showed significantly higher ratings than NIB-labelled products, particularly in terms of HP. These results were in line with findings from previous studies that demonstrated that Nutri-Score helped more consumers to identify the products as more valid from a nutritional point of view compared to other FoP labelling systems, probably encouraging them towards healthier food choices [27,35,61].

The ANOVA results showed that the healthiness perception decreased according to the NS category and hence according to the nutritional quality of the yoghurt: the dark green NS category “A” was perceived as significantly healthier than the light green NS category “B” and also the yellow NS category “C” (*p* < 0.05). This result confirmed the effectiveness of the Nutri-Score in helping consumers discriminate between products of different nutritional quality, since the progressive decrease in perceived healthiness from category “A” (dark green) to “C” (yellow) suggested that participants correctly interpreted the NS gradient. These findings were consistent with previous studies showing that NS facilitates consumers’ understanding and comparison of the overall nutritional value of foods [35,62,63]. However, in terms of willingness to buy, high and medium nutritional quality yoghurt showed no significantly different WTB values (*p* > 0.05), but it was significantly different from the low nutritional quality yoghurt (*p* < 0.05). In particular, yoghurts with a positive NS category (dark green “A” and light green “B”) received higher WTB scores than low nutritional quality yoghurt. The same trend, for both HP and WTB, was observed for NIB-labelled yoghurt. Therefore, it was possible to state that both FoP labelling systems were useful in guiding consumers towards healthier choices and both can help in boosting the purchasing of healthy products but not altering the purchase intention of consumers towards less healthy products, in accordance with results from De Temmerman et al. [63].

The pattern of results differed when considering fruit jam (Table 4). In this case, NIB-labelled fruit jams, particularly those with medium and low nutritional quality, received significantly higher scores than NS-labelled ones in both HP and WTB (*p* < 0.05). On the other hand, for high nutritional quality fruit jam, no significant differences emerged between NS and NIB for both HP and WTB (*p* > 0.05). This result was in agreement with previous studies which indicated that the impact of FoP labels on consumers’ experience can differ according to the nutritional profile of the product, with labelling effects often being more pronounced for products with low nutritional quality [64]. Taking into account that the main nutritional difference among fruit jams was their sugar content, the observed result could be explained by the fact that, nowadays, Italian consumers are increasingly aware of the adverse health effects associated with excessive sugar intake. This heightened awareness has likely been fostered by recent public health campaigns aimed at discouraging sugar consumption, as well as by the public debate surrounding the proposed Italian Sugar Tax [65]. Specifically, fruit jam labelled with orange NS category “D” was perceived as less healthy compared to the same product labelled with NIB. The NS classification, with its strong visual coding (letter “D” and orange color), was interpreted as a negative cue, leading to perceived healthiness values below the midpoint of the scale, thus categorizing the product as unhealthy. On the other hand, the NIB system, by displaying a battery icon for sugar that did not appear extreme (12% per portion), mitigated this perception, appearing less alarming to consumers compared to the same product labelled with the orange NS category “D”, thus resulting in a perceived healthiness score slightly above the midpoint of the scale. This could suggest that NS may have a stronger penalizing effect on products high in critical nutrients (i.e., sugar), while NIB gave an additional interpretation that allows consumers to make a less severe assessment.

Overall, these findings supported the growing body of literature highlighting that the effect of FoP labelling systems on consumers’ HP and WTB depends not only on the overall nutritional quality of the product, but also on the specific nutrient emphasized by the label [66]. In particular, previous studies showed that nutrients typically perceived as unhealthy—such as saturated fat, salt, and sugar—play a pivotal role in shaping healthiness perception by consumers, with negative FoP labels linked to high levels of these nutrients exerting a stronger impact than positive FoP labels highlighting their low amounts [67]. Moreover, previous studies also showed that there is a difference in healthfulness perception between directive and informative FoP labelling systems, and that this difference depends on the type of product. For example, for products with intermediate healthfulness scores (i.e., breakfast cereals, yoghurt) the impact of the FoP label was more pronounced, whereas it was less evident for products generally perceived as either completely healthy or entirely unhealthy [68]. Therefore, it was not possible to compare the results from different product categories, because each product involves a distinct consumer perception [63].

As regards differences among fruit jams according to their different nutritional quality, the trend was the same as that highlighted for yoghurts, but with a few differences. Indeed, based on the ANOVA results, for the NS-labelled fruit jams, the WTB values showed the same trend of HP, with high nutritional quality products showing significantly higher values than medium and also low nutritional quality fruit jams. This trend was not observed for NIB-labelled fruit jams, for which high and medium nutritional quality products showed no significantly different WTB values but were significantly different from low nutritional quality fruit jams.

Overall, these results suggested that FoP labels modulate the consumers’ healthiness perception, but their impacts were strongly context-dependent, varying across both product category and nutritional profile, possibly because both labels provided useful but different types of information. Therefore, these findings demonstrated that the two FoP labelling systems may be equally effective—or ineffective—in directly shaping consumers’ perceptions. Finally, these results provided partial support for hypothesis H1, indicating that the difference between NS and NIB depends on product category and nutritional quality level.

### 4.3. Predictors of Differences Among FoP Labels in Healthiness Perception and Willingness to Buy

The full results of all HMLR models separately applied to the 12 tested products, in order to explore the potential predictors of changes in healthiness perception (ΔHP) and willingness to buy (ΔWTB) between NS and NIB, are reported in the Supplementary Materials (Tables S3–S14).

**Figure 2:**
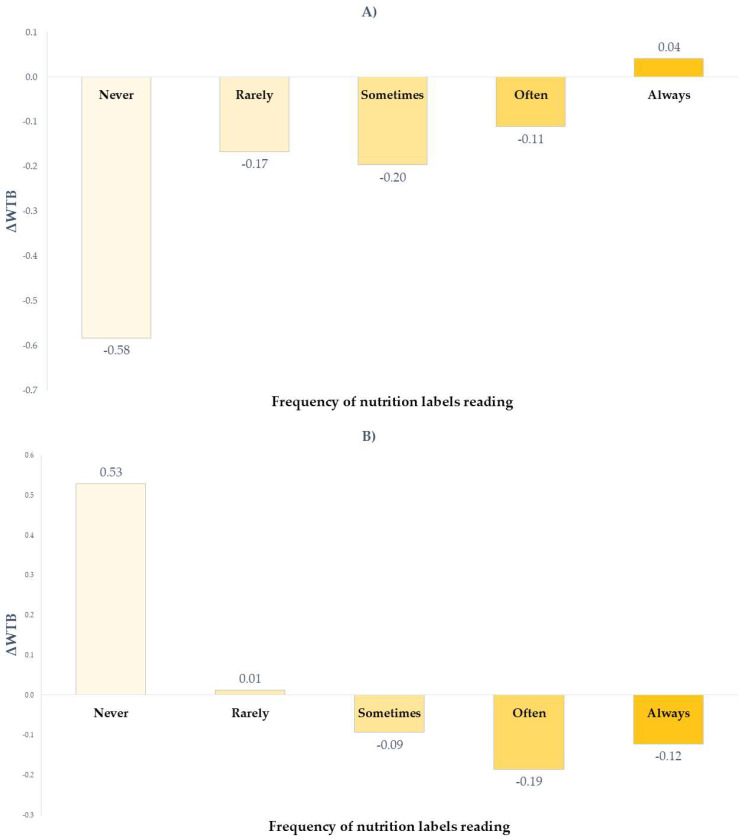
Effect of frequency of nutrition label reading on ΔWTB (WTB_NIB_ − WTB_NS_) of high (**A**) and low (**B**) nutritional quality yoghurt.

For yoghurt, the frequency of nutrition label reading emerged as a significant predictor of ΔWTB both for high and low nutritional quality levels. Specifically, for high nutritional quality yoghurt, ΔWTB increased with label reading frequency (*B* = 0.119, *p* = 0.047), meaning that individuals who more frequently read nutrition information were more likely to prefer NIB over the NS. As shown in Figure 2A, ΔWTB value increased from negative (−0.58) among participants who never read labels to slightly positive (+0.04) among those who always do, indicating that non-readers preferred the product with NS, whereas frequent label readers favored the same product when labelled with NIB. This pattern was reversed for low nutritional quality yoghurt (*B* = −0.144, *p* = 0.014). Indeed, as shown in Figure 2B, ΔWTB was positive among non-readers (+0.53) and turned negative among frequent readers (−0.12). This opposite trend suggested that the influence of FoP labels on consumers’ food choice may be moderated by consumers’ involvement with nutrition information. Participants who rarely consult food labels may tend to prefer the directive Nutri-Score label, which may elicit intuitive and faster judgments. A possible explanation of this result was that the modern consumers are often looking for ways to reduce time spent on food shopping, thus reducing the time necessary to take into consideration the information provided by the package [69]. Conversely, individuals who habitually read nutrition labels may be more familiar in processing detailed nutrient information and may prefer the NIB format, perceiving it as more informative and reliable. Indeed, as also emerged in previous studies, NIB was perceived by consumers as useful for understanding the product composition [24]. On the other hand, summary labels–such as NS–facilitate consumers in making food decisions in time-constrained situations [25]. Therefore, according to the results of this study, consumers who do not usually read labels were more attracted by the dark green NS category “A” logo than those who read and interpret all batteries of the NIB to process information related to the product. However, this could led to a *halo effect*, that is, a cognitive bias that leads people to generalize their opinion based on only one aspect of the product (the green label in the case of NS), without inquiring about the portions and frequencies of intake, resulting in over-consumption [6].

**Figure 3:**
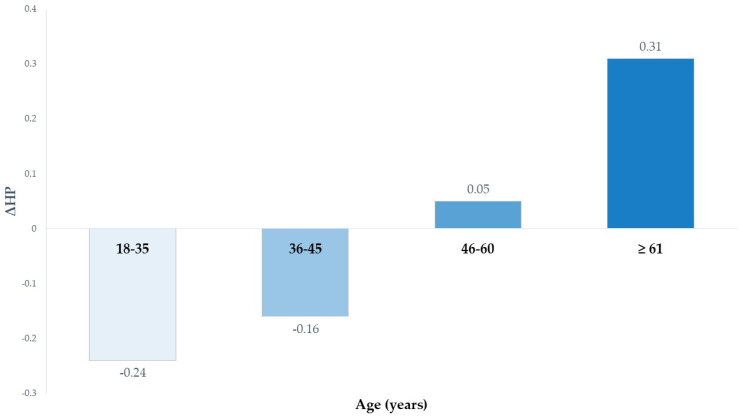
Effect of age on ΔHP (HP_NIB_ − HP_NS_) of high nutritional quality fruit jam.

For fruit jam of high nutritional quality, age emerged as a significant predictor. As shown in Figure 3, the ΔHP increased progressively with age (*B* = 0.149, *p* = 0.016) (from −0.24 among participants in the age range of 18–35 years to +0.31 among participants ≥ 61 years), indicating that older consumers tended to perceive NIB-labelled products as healthier than those labelled with NS. This result was probably explained because older individuals are generally more concerned about diet-related health outcomes [70] and hence may appreciate the more informative NIB nutrient-specific battery format, since it might allow older adults to have a more favorable interpretation of the product’s nutritional content. Therefore, older consumers more attentive to specific nutrient values may have a greater preference for the informative FoP labelling scheme, which is perceived as a health promotion intervention [71].

**Figure 4:**
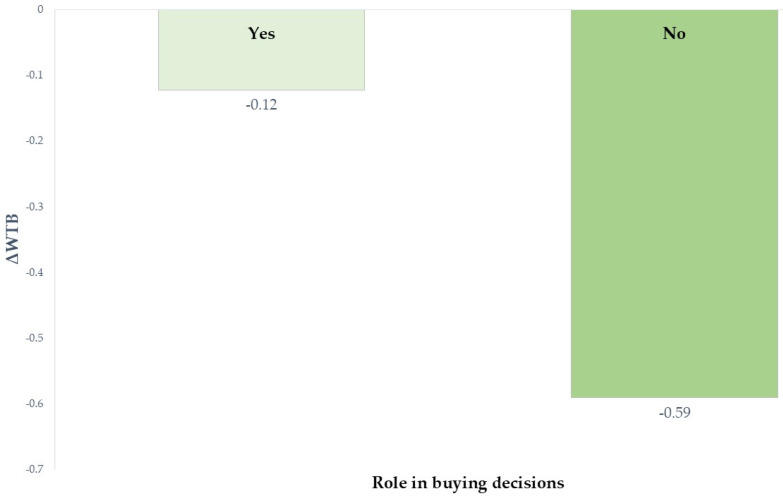
Effect of role in buying decisions on ΔWTB (WTB_NIB_− WTB_NS_) for high nutritional quality fruit jam.

A significant effect of the role of buying decisions was also found in ΔWTB for high nutritional quality fruit jam (*B* = −0.495, *p* = 0.019). As reported in Figure 4, both groups “Yes” (those primarily responsible for shopping) and “No” (including those sharing shopping responsibility, making suggestions, and those not responsible for shopping at all) showed negative ΔWTB values, thus indicating higher willingness to buy for NS-labelled products than NIB-labelled ones. However, the difference was more pronounced among participants who were not responsible for grocery shopping (ΔWTB = −0.59 vs. −0.12). This result suggested that direct exposure to purchasing contexts may moderate the influence of FoP labels. Indeed, consumers found it more difficult to comprehend the informative non-directive labelling system that provides information related to the name and amounts of nutrients in relation to total daily needs. Differentiating between healthy and unhealthy products based on the evaluation of nutrient intake may become complex tasks for those who are less involved in buying decisions [72]. Therefore, non-responsible shoppers, less familiar with product labels, might rely more on the intuitive color-coded system of NS, thus showing stronger differences between NS and NIB. On the other hand, individuals who regularly engage in food shopping could have more experience interpreting label information and may use it more critically, reducing the gap between directive (i.e., NS) and informative (i.e., NIB) FoP labelling systems. Overall, the regression analyses showed that, among sociodemographic characteristics, only age predicted ΔHP and ΔWTB, as well as the purchasing behaviors, thus offering partial support for hypothesis H2.

Although the educational level of the sample was relatively high, the study included cognitive constructs such as NfC and SAEI because these measures help in capturing consumers’ motivation and perceived competence in processing nutrition information [44]. Both in terms of HP and WTB, and for both product categories, no significant effect of orthorexia nervosa, NfC, and SAEI emerged in predicting the differences between the two FoP labelling systems. Although it was hypothesized that individuals with different levels of orthorexia nervosa, cognition need, and ability would show different preferences for the two different FoP label formats, this pattern did not emerge; therefore, hypothesis H3 was not supported. However, some non-significant trends were observed, thus aligning with the initial hypotheses. In particular, for high nutritional quality yoghurt, ON showed a tendency to influence ΔHP (*p* = 0.076), suggesting that individuals with higher concern for healthy eating may perceive NIB-labelled products as healthier than those labelled with NS. Therefore, the tendency toward orthorexic traits could make consumers more attracted by reductive FoP label formats, such as NIB. Probably, with a sample including participants with higher levels of orthorexia nervosa, this effect would become more evident. Neither NfC nor SAEI significantly predicted differences between FoP labels; thus, hypothesis H4 was not supported. Despite this, for low nutritional quality yoghurt, a slightly negative trend was observed between NfC and ΔWTB, even if non-significant (*p* = 0.060). This suggested that individuals with higher NfC were slightly less willing to buy NIB-labelled products compared to NS-labelled ones. Considering that the participants of this study showed low–moderate levels of cognitive engagement, this trend might be emphasized in groups characterized by higher NfC scores. However, given that these tendencies emerged only for yoghurt and not for fruit jam, these findings suggested the pivotal role of product category in shaping consumers’ responses to FoP labels.

### 4.4. Limitations and Future Outlook

The main limitations of this study were the restricted range of product categories and self-reported consumers’ responses based on survey data. Future research should expand the scope to include products with poorer nutritional qualities and belonging to diverse product categories and should assess consumers’ responses in real-life purchasing contexts, such as supermarkets or online shopping environments, so as to explore the actual consumers’ purchasing behaviors.

Secondly, the participants of this study showed limited heterogeneity in orthorexia nervosa, Need for Cognition, and Subjective Ability to Evaluate Information, although showing high educational level. This lack of variability may have reduced the likelihood of identifying significant effects of these traits in consumers’ responses to FoP labels; probably, the limited variability in formal education in the sample may have reduced the potential to detect stronger moderating effects of cognitive abilities on consumers’ decisions. Therefore, future studies with a larger and more heterogeneous sample of participants, with broader ranges of orthorexic tendencies and cognitive abilities, could help clarify these effects. Combining self-reported data, such as the FoP labels perception assessment used in this study, with implicit measures, such as the eye-tracking method, may provide a more comprehensive understanding of how consumers process and interpret FoP labels.

A further limitation concerns the representativeness of the sample, which included mainly women and younger adults from northern Italy. Although women are typically responsible for the majority of household food purchasing, managing almost 80% of domestic expenditure [73], and younger individuals are highly engaged with nutrition information, these characteristics limited the generalizability of the findings to the broader Italian population. Future online studies should therefore adopt sampling methods ensuring a more balanced distribution across gender, age groups, and geographic areas.

## 5. Conclusions

Overall, the findings of this study partially supported the proposed initial hypotheses. Significant differences in consumers’ healthiness perceptions and willingness to buy between the two FoP labelling schemes—Nutri-Score vs. NutrInform Battery—were observed, although the direction and the magnitude of these effects varied across product categories and nutritional quality.

Sociodemographic and purchasing behavioral variables such as age, role in buying decisions, and frequency of nutritional label reading emerged as significant predictors of consumers’ responses to FoP labels. Older participants and those who frequently read nutrition information tended to prefer the NutrInform Battery format, particularly for high nutritional quality products. Conversely, individuals less involved in grocery shopping or less focused on food labels showed a greater preference for Nutri-Score, especially when applied to products of higher nutritional quality.

Although the tendency toward orthorexia nervosa (measured through the Eating Habits Questionnaire-21) did not significantly predict differences in consumers’ HP and WTB between NS and NIB, a non-significant trend suggested that higher orthorexic tendencies might be associated with preference for the NIB format for high nutritional quality yoghurt. Similarly, although cognitive abilities—Need for Cognition and Subjective Ability to Evaluate Information—were not able to predict ΔHP and ΔWTB, a slightly negative trend suggested that individuals with higher NfC tended to prefer the NS format.

In conclusion, these findings provided several practical implications for policymakers and stakeholders involved in the implementation of FoP labelling systems, thus contributing to the ongoing policy debate on FoP label harmonization in Europe and suggesting that adopting either a purely directive or purely reductive approach may not be appropriate. Indeed, this study suggested that a uniform FoP label may not be equally beneficial for the entire population. Therefore, insights from this study supported a more consumer-centric approach to FoP policy design, recommending flexibility and co-creation strategies, involving both policymakers, industries, and consumers, in order to propose FoP label formats that effectively guide healthier food choices while accommodating diverse consumers’ cognitive profiles and information-processing styles.

## Figures and Tables

**Table 1:** Experimental conditions evaluated in the survey.

Product Category	Front-of-Pack Label	Nutritional Quality		
		High	Medium	Low
**Yoghurt**	Nutri-Score	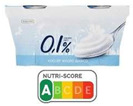	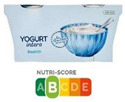	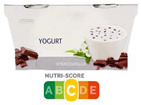
	NutrInform Battery	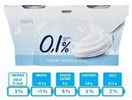	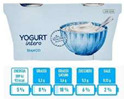	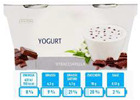
**Fruit jam**	Nutri-Score	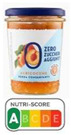	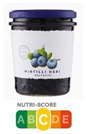	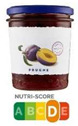
	NutrInform Battery	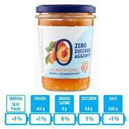	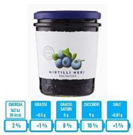	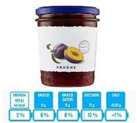

**Table 2:** Sociodemographic characteristics and purchasing behaviors of the survey participants (*n* = 436).

Variable	*N*	%
***Gender***		
Female	309	70.87
Male	124	28.44
Other	1	0.23
I prefer not to declare it	2	0.46
***Age***		
18–35	217	49.77
36–45	76	17.43
46–60	97	22.25
≥61	46	10.55
***Education level***		
Elementary school diploma	1	0.23
Middle school diploma	7	1.61
High school diploma (or equivalent)	96	22.02
Bachelor’s degree	90	20.64
Postgraduate degree (master’s, doctorate)	242	55.50
Area of residence *		
Northwest	278	64.80
Northeast	48	11.19
Center	24	5.59
South	70	16.32
Island	9	2.10
***Diet***		
Omnivorous	338	77.52
Flexitarian	70	16.06
Vegetarian	25	5.73
Vegan	3	0.69
***Role in buying decisions***		
I am primarily responsible for these purchases	194	44.50
I am not involved in the decision at all	6	1.38
I make suggestions but decisions are made by other family members	50	11.47
I share the responsibility for making these purchases	186	42.66
***Reading of nutritional labels***		
Never	42	9.63
Rarely	92	21.10
Sometimes	113	25.92
Often	140	32.11
Always	49	11.24

* Classification according to Nomenclature of Territorial Units for Statistics (NUTS).

**Table 3:** Differences between Nutri-Score (NS)- and NutrInform Battery (NIB)-labelled yoghurts in healthiness perception (HP) and willingness to buy (WTB), including differences across nutritional quality levels for each Front-of-Pack label.

Product Category	Nutritional Quality	HP			WTB		
		NS	NIB	p-Value *	NS	NIB	p-Value *
**Yoghurt**	**High**	5.6 ± 1.5 ^a^	5.3 ± 1.6 ^a^	***0.004***	5.1 ± 1.8 ^a^	4.9 ± 1.9 ^a^	*ns*
	**Medium**	5.2 ± 1.4 ^b^	4.8 ± 1.6 ^b^	***0.001***	5.0 ± 1.7 ^a^	4.7 ± 1.8 ^a^	***0.019***
	**Low**	3.7 ± 1.4 ^c^	3.6 ± 1.6 ^c^	*ns*	3.8 ± 1.8 ^b^	3.7 ± 2.0 ^b^	*ns*
	p-value **	***<0.001***	***<0.001***		***<0.001***	***<0.001***	

Different letters in the same column indicate statistically significant difference according to Tukey’s post hoc test (*p* < 0.05). ns = not significant. * *p*-value of the two-sample paired *t*-tests; ** *p*-value of the two-ways ANOVA.

**Table 4:** Differences between Nutri-Score (NS)- and NutrInform Battery (NIB)-labelled fruit jams in healthiness perception (HP) and willingness to buy (WTB), including differences across nutritional quality levels for each Front-of-Pack label.

Product Category	Nutritional Quality	HP			WTB		
		NS	NIB	p-Value *	NS	NIB	p-Value *
**Fruit jam**	**High**	5.0 ± 1.6 ^a^	4.9 ± 1.5 ^a^	*ns*	4.9 ± 1.8 ^a^	4.7 ± 1.8 ^a^	*ns*
	**Medium**	4.1 ± 1.3 ^b^	4.4 ± 1.5 ^b^	***0.004***	4.3 ± 1.7 ^b^	4.5 ± 1.8 ^a^	*0.033*
	**Low**	3.5 ± 1.5 ^c^	4.1 ± 1.5 ^c^	***<0.0001***	3.6 ± 1.8 ^c^	4.1 ± 1.8 ^b^	***<0.0001***
	p-value **	***<0.001***	***<0.001***		***<0.001***	***<0.001***	

Different letters in the same column indicate statistically significant difference according to Tukey’s post hoc test (*p* < 0.05). ns = not significant. * *p*-value of the two-sample paired *t*-tests; ** *p*-value of the two-ways ANOVA.

## Data Availability

The original contributions presented in the study are included in the article/Supplementary Material, further inquiries can be directed to the corresponding author.

## Supplementary Materials

The following supporting information can be downloaded at: https://www.mdpi.com/article/10.3390/foods14234033/s1. Table S1. Descriptive statistics and reliability analysis of the orthorexia nervosa scale (1 = strongly disagree, 7 = strongly agree). Table S2. Descriptive statistics and reliability analysis of the scales related to cognitive abilities (1 = strongly disagree, 7 = strongly agree). Table S3. Hierarchical Multiple Linear Regression models explaining difference between NutrInform Battery and Nutri-Score in healthiness perception (ΔHP) of high nutritional quality yoghurt. Table S4. Hierarchical Multiple Linear Regression models explaining difference between NutrInform Battery and Nutri-Score in healthiness perception (ΔHP) of medium nutritional quality yoghurt. Table S5. Hierarchical Multiple Linear Regression models explaining difference between NutrInform Battery and Nutri-Score in healthiness perception (ΔHP) of low nutritional quality yoghurt. Table S6. Hierarchical Multiple Linear Regression models explaining difference between NutrInform Battery and Nutri-Score in willingness to buy (ΔWTB) of high nutritional quality yoghurt. Table S7. Hierarchical Multiple Linear Regression models explaining difference between NutrInform Battery and Nutri-Score in willingness to buy (ΔWTB) of medium nutritional quality yoghurt. Table S8. Hierarchical Multiple Linear Regression models explaining difference between NutrInform Battery and Nutri-Score in willingness to buy (ΔWTB) of low nutritional quality yoghurt. Table S9. Hierarchical Multiple Linear Regression models explaining difference between NutrInform Battery and Nutri-Score in healthiness perception (ΔHP) of high nutritional quality jam. Table S10. Hierarchical Multiple Linear Regression models explaining difference between NutrInform Battery and Nutri-Score in healthiness perception (ΔHP) of medium nutritional quality jam. Table S11. Hierarchical Multiple Linear Regression models explaining difference between NutrInform Battery and Nutri-Score in healthiness perception (ΔHP) of low nutritional quality jam. Table S12. Hierarchical Multiple Linear Regression models explaining difference between NutrInform Battery and Nutri-Score in willingness to buy (ΔWTB) of high nutritional quality jam. Table S13. Hierarchical Multiple Linear Regression models explaining difference between NutrInform Battery and Nutri-Score in willingness to buy (ΔWTB) of medium nutritional quality jam. Table S14. Hierarchical Multiple Linear Regression models explaining difference between NutrInform Battery and Nutri-Score in willingness to buy (ΔWTB) of low nutritional quality jam.
