# Supplementary material for: Directive vs. Reductive Front-of-Pack Labels: Differences in Italian Consumers’ Responses to the Nutri-Score and the NutrInform Battery
Source: Foods. 2025 Nov 25;14(23):4033. doi: 10.3390/foods14234033 (PMC12691927; doi:10.3390/foods14234033)
Supplement: Supplementary file 1 [file foods-14-04033-s001.zip › FoP label survey_supplementary.pdf]

Start of Block: INTRODUZIONE

DESCRIZIONE GENERALE DELLO STUDIO
Gentile Partecipante, la ringraziamo per il suo interesse verso questo studio condotto da un team di ricercatori dell'**Università di Scienze Gastronomiche di Pollenzo** (www.unisg.it) e dell'**Università degli Studi di Pavia** (www.unipv.it).
Il questionario a cui sta per rispondere fa parte del progetto NODES, sostenuto dal MUR sui fondi PNRR MUR - M4C2 - Investimento 1.5 Avviso "Ecosistemi dell'Innovazione", nell'ambito del PNRR finanziato dall'Unione europea – NextGenerationEU (Grant agreement Cod. n.ECS00000036).
Questo studio è volto ad indagare l'attitudine dei consumatori nei confronti delle etichette nutrizionali e come esse ne influenzano le scelte alimentari. In questo questionario, le verrà chiesto di rispondere ad una serie di domande riguardo a tratti della personalità, abitudini alimentari, caratteristiche sociodemografiche che influenzano la comprensione delle informazioni riportate in etichetta, al fine di indentificare quale sistema di etichettatura risulta più efficace nel comunicare in maniera chiara e comprensibile la qualità nutrizionale dei prodotti alimentari.
La compilazione del questionario richiede circa **13-15 minuti**.
La partecipazione allo studio è completamente volontaria. È possibile ritirarsi in qualsiasi momento.
CONSENSO INFORMATO
Tutti i dati dello studio sono registrati in forma anonima e trattati in forma aggregata, ai sensi della legge n.196/03 ed emendamenti (pubblicati sulla Gazzetta Ufficiale n.65, 19 Marzo 2007 codice in materia di protezione dei dati personali) e del Reg. (EU) 679/2016.
Questo studio è stato approvato dal Comitato Etico dell'Università di Scienze Gastronomiche di Pollenzo (Verbale n. 2023.04). Tutti i dati saranno utilizzati esclusivamente dai ricercatori a fini di ricerca, divulgazione e pubblicazioni scientifiche.
Per ulteriori informazioni su questo questionario, può contattare il referente dello studio:
Dott.ssa Nazarena Cela (n.cela@unisg.it)
Grazie ancora per la disponibilità a partecipare a questo studio!

Se ha un'età superiore ai 18 anni e vuole partecipare allo studio, fornisca il suo consenso (necessario per proseguire):

○ **Do il mio consenso** (1)

○ Non do il mio consenso (2)

End of Block: INTRODUZIONE

Start of Block: Comportamento all' acquisto

Quale delle seguenti affermazioni descrive meglio il suo ruolo quando si tratta di acquistare generi alimentari per la sua famiglia?

- ◯ Sono il principale responsabile per questi acquisti (1)
- ◯ Condivido la responsabilità di fare questi acquisti (2)
- ◯ Fornisco suggerimenti ma le decisioni sono prese da altri membri della famiglia (3)
- ◯ Non sono affatto coinvolto nella decisione (4)

Prima dell'acquisto di generi alimentari controlla la tabella nutrizionale?

- ◯ Mai (1)
- ◯ Raramente (2)
- ◯ A volte (3)
- ◯ Spesso (4)
- ◯ Sempre (5)

End of Block: Acquisto

Start of Block: Frequenza di consumo

Quanto frequentemente consuma yogurt?

- Mai (1)
- <1/mese (2)
- 1–3/mese (3)
- 1–2/settimana (4)
- 3–4/settimana (5)
- 5–6/settimana (6)
- 1/giorno (7)
- 2+/giorno (8)

Quanto frequentemente consuma confetture?

- Mai (1)
- <1/mese (2)
- 1–3/mese (3)
- 1–2/settimana (4)
- 3–4/settimana (5)
- 5–6/settimana (6)
- 1/giorno (7)
- 2+/giorno (8)

End of Block: Frequenza di consumo

Start of Block: FoP

Utilizzando la scala da 1 a 7 punti, indichi la sua intenzione all'acquisto e la percezione di salubrità dei prodotti che le verranno mostrati di seguito, tenendo in considerazione **l'informazione nutrizionale associata al prodotto.**
Intenzione all'acquisto: 1= non lo comprerei affatto; 7= sicuramente lo comprerei
Percezione di salubrità: 1=non salutare; 7= salutare

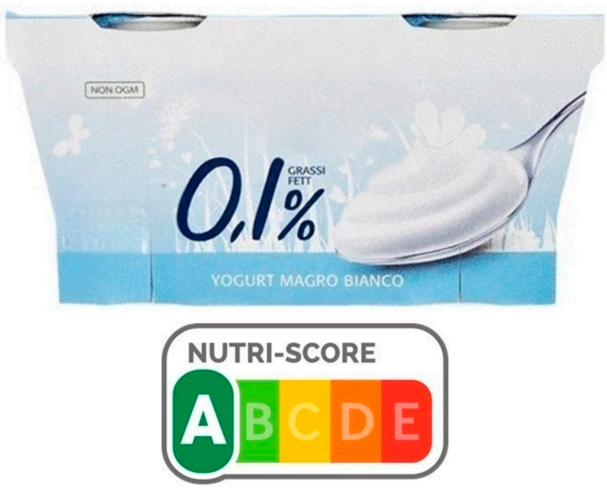

	1 (1)	2 (2)	3 (3)	4 (4)	5 (5)	6 (6)	7 (7)	
1. Non lo comprerei affatto	○	○	○	○	○	○	○	7. Sicuramente lo comprerei
1. Non salutare	○	○	○	○	○	○	○	7. Salutare

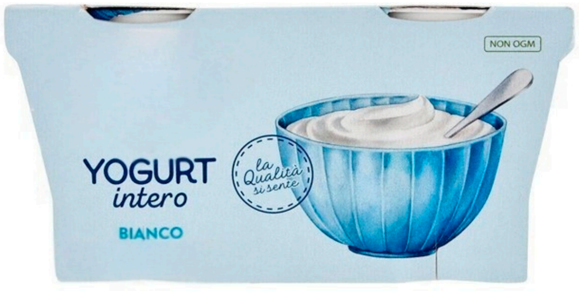

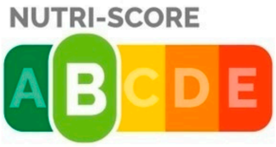

	1 (1)	2 (2)	3 (3)	4 (4)	5 (5)	6 (6)	7 (7)	
1. Non lo comprerei affatto	○	○	○	○	○	○	○	7. Sicuramente lo comprerei
1. Non salutare	○	○	○	○	○	○	○	7. Salutare

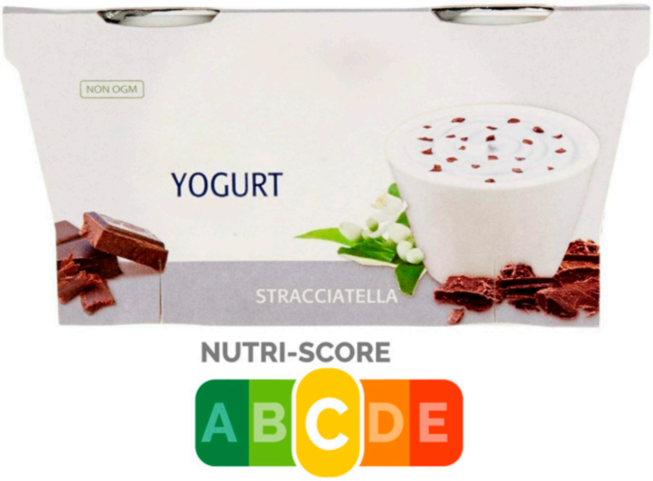

	1 (1)	2 (2)	3 (3)	4 (4)	5 (5)	6 (6)	7 (7)	
1. Non lo comprerei affatto	○	○	○	○	○	○	○	7. Sicuramente lo comprerei
1. Non salutare	○	○	○	○	○	○	○	7. Salutare

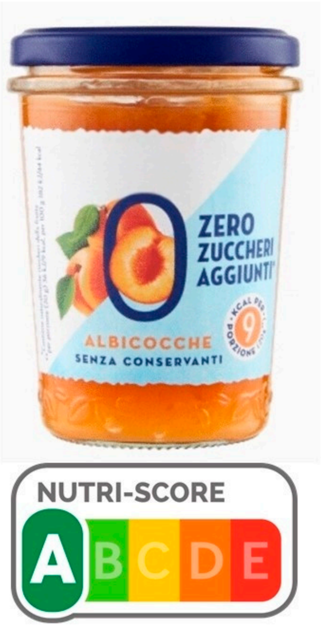

	1 (1)	2 (2)	3 (3)	4 (4)	5 (5)	6 (6)	7 (7)	
1. Non lo comprerei affatto	○	○	○	○	○	○	○	7. Sicuramente lo comprerei
1. Non salutare	○	○	○	○	○	○	○	7. Salutare

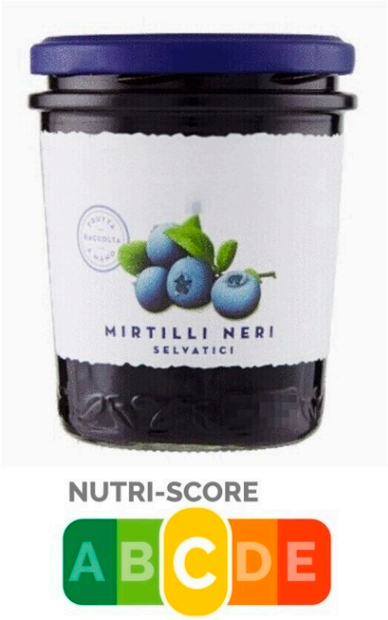

	1 (1)	2 (2)	3 (3)	4 (4)	5 (5)	6 (6)	7 (7)	
1. Non lo comprerei affatto	○	○	○	○	○	○	○	7. Sicuramente lo comprerei
1. Non salutare	○	○	○	○	○	○	○	7. Salutare

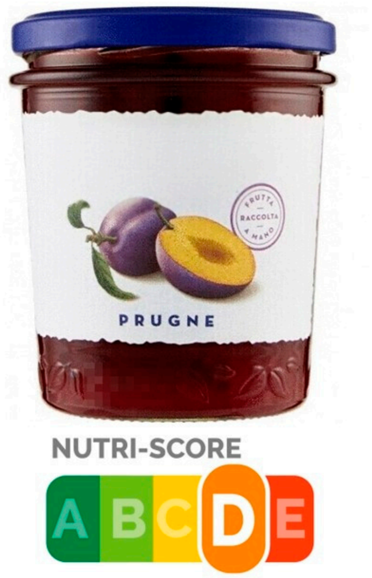

	1 (1)	2 (2)	3 (3)	4 (4)	5 (5)	6 (6)	7 (7)	
1. Non lo comprerei affatto	○	○	○	○	○	○	○	7. Sicuramente lo comprerei
1. Non salutare	○	○	○	○	○	○	○	7. Salutare

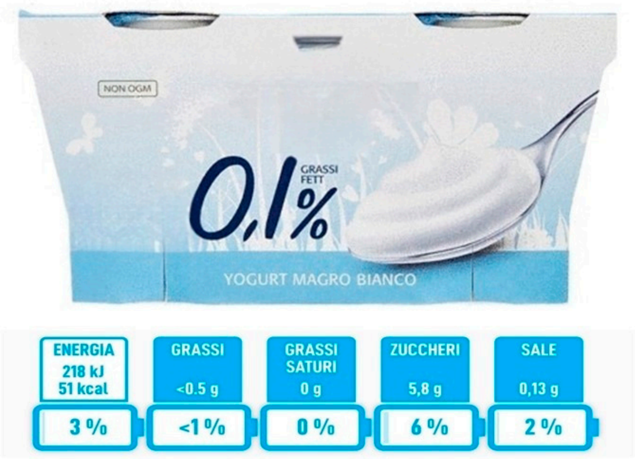

	1 (1)	2 (2)	3 (3)	4 (4)	5 (5)	6 (6)	7 (7)	
1. Non lo comprerei affatto	○	○	○	○	○	○	○	7. Sicuramente lo comprerei
1. Non salutare	○	○	○	○	○	○	○	7. Salutare

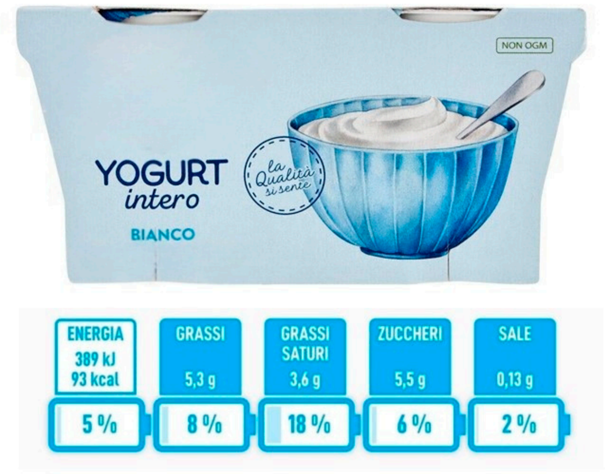

	1 (1)	2 (2)	3 (3)	4 (4)	5 (5)	6 (6)	7 (7)	
1. Non lo comprerei affatto	○	○	○	○	○	○	○	7. Sicuramente lo comprerei
1. Non salutare	○	○	○	○	○	○	○	7. Salutare

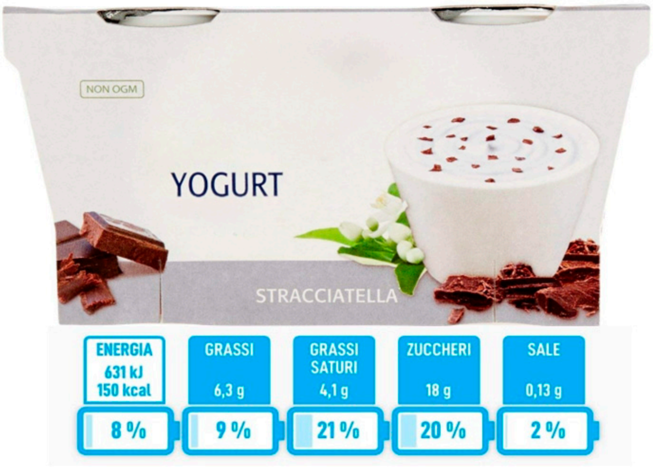

	1 (1)	2 (2)	3 (3)	4 (4)	5 (5)	6 (6)	7 (7)	
1. Non lo comprerei affatto	○	○	○	○	○	○	○	7. Sicuramente lo comprerei
1. Non salutare	○	○	○	○	○	○	○	7. Salutare

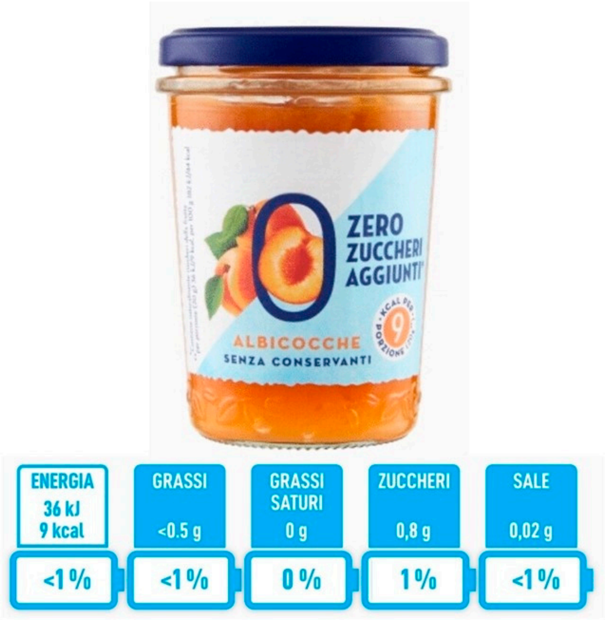

	1 (1)	2 (2)	3 (3)	4 (4)	5 (5)	6 (6)	7 (7)	
1. Non lo comprerei affatto	○	○	○	○	○	○	○	7. Sicuramente lo comprerei
1. Non salutare	○	○	○	○	○	○	○	7. Salutare

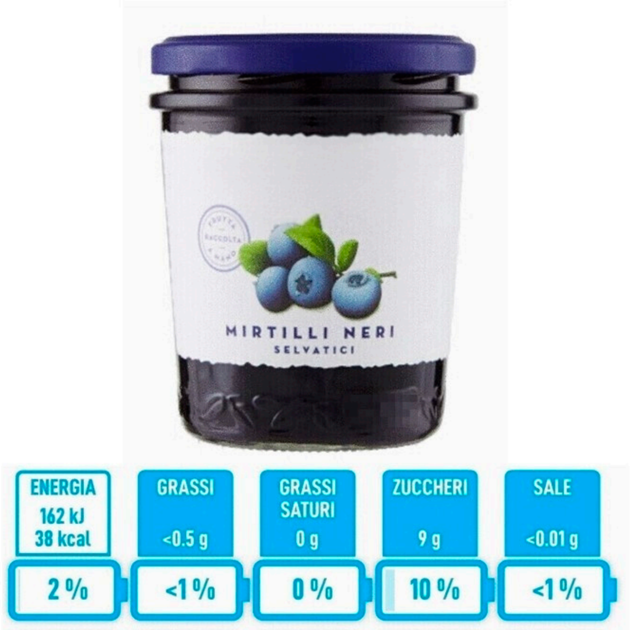

	1 (1)	2 (2)	3 (3)	4 (4)	5 (5)	6 (6)	7 (7)	
1. Non lo comprerei affatto	○	○	○	○	○	○	○	7. Sicuramente lo comprerei
1. Non salutare	○	○	○	○	○	○	○	7. Salutare

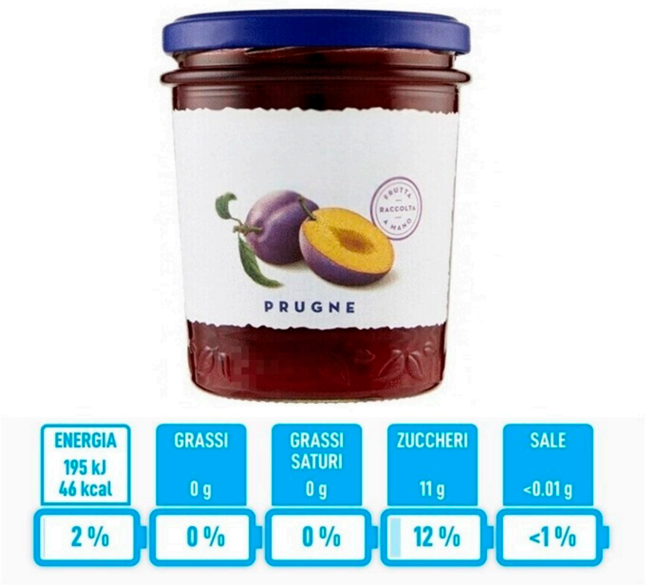

	1 (1)	2 (2)	3 (3)	4 (4)	5 (5)	6 (6)	7 (7)	
1. Non lo comprerei affatto	○	○	○	○	○	○	○	7. Sicuramente lo comprerei
1. Non salutare	○	○	○	○	○	○	○	7. Salutare

End of Block: FoP

Start of Block: EHQ-21

Per ognuna delle seguenti affermazioni, indichi il livello di accordo con punteggio da 1 “totalmente in disaccordo” a 7 “totalmente d’accordo”

	1. Totalmente in disaccordo (1)	2 (2)	3 (3)	4 (4)	5 (5)	6 (6)	7. Totalmente d'accordo (7)
Sono molto più informato di altri riguardo l’alimentazione sana (1)	o	o	o	o	o	o	o
Rinuncio a impegni sociali che comportano un’alimentazione malsana (2)	o	o	o	o	o	o	o
Il modo in cui è preparato il mio cibo è importante nella mia dieta (3)	o	o	o	o	o	o	o
Seguo una dieta con molte regole (4)	o	o	o	o	o	o	o
Le mie abitudini alimentari sono migliori di quelle degli altri (5)	o	o	o	o	o	o	o
Sono distratto da pensieri che riguardano il mangiare sano (6)	o	o	o	o	o	o	o
Mangio solo ciò che la mia dieta consente (7)	o	o	o	o	o	o	o
Il mio modo salutare di mangiare è un’importante fonte di problemi nelle mie relazioni (8)	o	o	o	o	o	o	o

Ho dovuto fare sforzi nel corso del tempo per mangiare più sano (9)							
La mia dieta influisce sul tipo di lavoro che potrei accettare (10)							
La mia dieta è meglio di quella di altre persone (11)							
Sento di avere il controllo quando mangio cibo salutare (12)							
Lo scorso anno, i miei amici o i familiari mi hanno detto che sono eccessivamente preoccupato riguardo il mangiare sano (13)							
Ho difficoltà a trovare ristoranti dove viene servito il cibo che mangio io (14)							
Mangiare come faccio io mi dà un senso di soddisfazione (15)							
Pochi alimenti sono salutari per me (16)							

Esco meno da quando ho iniziato a mangiare in modo salutare (17)	○	○	○	○	○	○	○
Passo più di tre ore al giorno pensando ad alimenti salutari (18)	○	○	○	○	○	○	○
Mi sento magnificamente quando mangio cibo sano (19)	○	○	○	○	○	○	○
Seguo rigidamente una dieta basata su alimenti sani (20)	○	○	○	○	○	○	○
Preparo i pasti nel modo più salutare possibile (21)	○	○	○	○	○	○	○

End of Block: EHQ-21

Start of Block: NfC and SAEI

Per ognuna delle seguenti affermazioni, indichi il livello di accordo con punteggio da 1 “totalmente in disaccordo” a 7 “totalmente d’accordo”

	1. Totalmente in disaccordo (1)	2 (2)	3 (3)	4 (4)	5 (5)	6 (6)	7. Totalmente d'accordo (7)
Preferirei problemi complessi a problemi semplici (1)	○	○	○	○	○	○	○
Mi piace avere la responsabilità di gestire una situazione che richiede molta riflessione (2)	○	○	○	○	○	○	○
Trovo soddisfazione nel riflettere duramente e per lunghe ore (3)	○	○	○	○	○	○	○
L'idea di affidarmi al pensiero per arrivare al top mi attrae (4)	○	○	○	○	○	○	○
Mi piace davvero il compito di trovare nuove soluzioni ai problemi (5)	○	○	○	○	○	○	○
Preferisco che la mia vita sia piena di rompicapi che devo risolvere (6)	○	○	○	○	○	○	○
La nozione di pensare in modo astratto mi attira (7)	○	○	○	○	○	○	○

Preferirei un compito intellettuale, difficile e importante rispetto ad uno che è allo stesso modo importante ma non richiede molta riflessione (8)	○	○	○	○	○	○	○
Di solito finisco per deliberare su questioni anche quando non mi riguardano personalmente (9)	○	○	○	○	○	○	○

Per ognuna delle seguenti affermazioni, indichi il suo livello di accordo con punteggio da 1 “totalmente in disaccordo” a 7 “totalmente d’accordo”

	1. Totalmente in disaccordo (1)	2 (2)	3 (3)	4 (4)	5 (5)	6 (6)	7. Totalmente d'accordo (7)
Per me è facile distinguere tra informazioni valide e non valide sulla salubrità delle scelte alimentari (1)	o	o	o	o	o	o	o
Per me è facile distinguere tra informazioni valide e non valide sull'impatto climatico delle scelte alimentari (2)	o	o	o	o	o	o	o
Per me è facile individuare fonti di informazioni affidabili e inaffidabili relative alla salubrità e al rispetto del clima degli alimenti (3)	o	o	o	o	o	o	o

End of Block: NfC and SAEI

Start of Block: Sociodemographic information

Genere

- Maschio (1)
- Femmina (2)
- Altro (3)
- Preferisco non dichiararlo (4)

Età

▼ 18 (1) ... 100 (83)

Regione in cui vivi

▼ Abruzzo (1) ... Veneto (20)

Q49 Livello di istruzione

- Licenza elementare (1)
- Diploma di scuola media inferiore (2)
- Diploma di scuola media superiore (o equivalente) (3)
- Laurea triennale (4)
- Titolo post-laurea (laurea magistrale, a ciclo unico, master, dottorato) (5)

Dieta alimentare

○ Onnivoro (1)

○ Flexitariano (2)

○ Vegetariano (3)

○ Vegano (4)

End of Block: Socio-dem
