# Supplementary material for: Directive vs. Reductive Front-of-Pack Labels: Differences in Italian Consumers’ Responses to the Nutri-Score and the NutrInform Battery
Source: Foods. 2025 Nov 25;14(23):4033. doi: 10.3390/foods14234033 (PMC12691927; doi:10.3390/foods14234033)
Supplement: Supplementary file 1 [file foods-14-04033-s001.zip › Supplementary materials_FoP label survey.pdf]

Supplementary materials

Table S1. Descriptive statistics and reliability analysis of the orthorexia nervosa scale (1= strongly disagree, 7 = strongly agree).

Scale	Item	Subscale	Subscale mean* (SD)	Item mean (SD)	Cronbach's α
Eating Habits Questionnaire-21	I am much more informed than others about healthy eating	Knowledges	4.7[a] (0.6)	4.9 (1.7)	0.818
	The way my food is prepared is important in my diet			5.4 (1.6)	
	My eating habits are better than those of others			4.2 (1.6)	
	My diet is better than other people's diets			4.0 (1.8)	
	I prepare meals in the healthiest way possible			4.8 (1.7)	
	I give up social commitments that involve unhealthy eating habits	Problems	2.4[c] (0.5)	2.9 (1.8)	0.874
	I follow a diet with many rules			3.4 (1.8)	
	I am distracted by thoughts about healthy eating			3.0 (1.7)	
	I only eat what my diet allows			2.6 (1.7)	
	My healthy eating habits are a major source of problems in my relationships			2.0 (1.5)	
	My diet affects the type of work I could take on			2.1 (1.6)	
	Last year, my friends or family members told me that I am overly concerned about healthy eating			2.1 (1.6)	
	I have difficulty finding restaurants where they serve the food that I eat			2.2 (1.7)	
	Few foods are healthy for me			2.3 (1.6)	
	I go out less since I started eating healthy			2.2 (1.6)	
	I spend more than three hours a day thinking about healthy foods			1.7 (1.2)	
	I strictly follow a diet based on healthy foods			2.7 (1.7)	
	I have had to make efforts over time to eat healthier	Feelings	4.3[b] (0.5)	3.7 (1.9)	0.648
	I feel in control when I eat healthy food			4.1 (1.8)	
	Eating the way I do gives me a sense of satisfaction			4.8 (1.7)	
	I feel great when I eat healthy food			4.4 (1.8)	

*Different letters in the same column indicate significant difference, according to Tukey's post-hoc test ($p < 0.05$).
SD = standard deviation.

Table S2. Descriptive statistics and reliability analysis of the scales related to cognitive abilities (1= strongly disagree, 7 = strongly agree).

Scale	Item	Scale mean (SD)	Item mean (SD)	Cronbach's α
Need for Cognition	I would prefer complex to simple problems	3.7 (0.6)	2.9 (1.8)	0.878
	I like to have the responsibility of handling a situation that requires a lot of thinking		4.2 (1.6)	
	I find satisfaction in deliberating hard and for long hours		3.3 (1.7)	
	The idea of relying on thought to make my way to the top appeals to me		4.0 (1.7)	
	I really enjoy a task that involves coming up with new solutions to problems		4.9 (1.6)	
	I prefer my life to be filled with puzzles that I must solve		3.0 (1.7)	
	The notion of thinking abstractly is appealing to me		3.6 (1.8)	
	I would prefer a task that is intellectual, difficult, and important to one that is somewhat important but does not require much thought		3.8 (1.6)	
	I usually end up deliberating about issues even when they do not affect me personally		3.8 (1.8)	
Subjective Ability to Evaluate Information	It is easy for me to distinguish between valid and invalid information about the healthiness of food choices	4.5 (0.2)	4.7 (1.6)	0.847
	It is easy for me to distinguish between valid and invalid information about the climate impacts of food choices		4.3 (1.6)	
	It is easy for me to identify reliable and unreliable information sources related to the healthiness and climate-friendliness of food		4.5 (1.6)	

SD = standard deviation.

Table S3. Hierarchical Multiple Linear Regression models explaining difference between NutrInform Battery and Nutri-Score in healthiness perception (ΔHP) of high nutritional quality yoghurt.

ΔHP_High nutritional quality yoghurt	Variables	Unstandardized coefficients (*B*)	Std.Error B	β	Sign.	R^2	F	Sign. Model
**Step 1**	(Constant)	-0.015	0.384		0.969	0.002	0.257	0.856
Sociodemographic	Gender	-0.024	0.136	-0.009	0.858			
	Age range	0.008	0.063	0.006	0.897			
	Educational level	-0.065	0.076	-0.042	0.395			
**Step 2**	(Constant)	-0.284	0.556		0.609	0.006	0.470	0.799
Sociodemographic	Gender	0.006	0.138	0.002	0.965			
	Age range	0.015	0.065	0.012	0.821			
	Educational level	-0.074	0.077	-0.048	0.341			
Purchasing behaviour	Role in buying decisions	0.016	0.217	0.004	0.943			
	Frequency of nutrition labels reading	0.075	0.060	0.064	0.213			
**Step 3**	(Constant)	-0.683	0.598		0.254	0.013	0.921	0.479
Sociodemographic	Gender	-0.010	0.138	-0.004	0.942			
	Age range	0.018	0.065	0.014	0.783			
	Educational level	-0.059	0.077	-0.039	0.443			
Purchasing behaviour	Role in buying decisions	0.048	0.217	0.011	0.826			
	Frequency of nutrition labels reading	0.027	0.065	0.023	0.677			
Orthorexia nervosa	Eating Habits Questionnaire-21	0.007	0.004	0.098	0.076			
**Step 4**	(Constant)	-0.810	0.609		0.184	0.018	0.899	0.518
Sociodemographic	Gender	-0.033	0.139	-0.012	0.814			
	Age range	0.023	0.065	0.018	0.727			
	Educational level	-0.062	0.079	-0.041	0.432			
Purchasing behaviour	Role in buying decisions	0.027	0.218	0.006	0.902			
	Frequency of nutrition labels reading	0.036	0.068	0.031	0.596			
Orthorexia nervosa	Eating Habits Questionnaire-21	0.006	0.004	0.081	0.159			
Cognitive abilities	Need for Cognition	0.008	0.006	0.067	0.198			
	Subjective Ability to Evaluate Information	-0.003	0.018	-0.010	0.861			

Table S4. Hierarchical Multiple Linear Regression models explaining difference between NutrInform Battery and Nutri-Score in healthiness perception (ΔHP) of medium nutritional quality yoghurt.

**ΔHP_Medium nutritional quality yoghurt**	**Variables**	**Unstandardized coefficients (*B*)**	**Std.Error B**	**β**	**Sign.**	**R^2**	**F**	**Sign. Model**
**Step 1**	(Constant)	0.088	0.367		0.811	0.008	1.046	0.372
Sociodemographic	Gender	-0.085	0.129	-0.032	0.513			
	Age range	0.060	0.060	0.050	0.318			
	Educational level	-0.099	0.073	-0.067	0.174			
**Step 2**	(Constant)	-0.511	0.530		0.336	0.014	1.119	0.350
Sociodemographic	Gender	-0.061	0.131	-0.023	0.642			
	Age range	0.080	0.062	0.066	0.194			
	Educational level	-0.091	0.074	-0.062	0.217			
Purchasing behaviour	Role in buying decisions	0.276	0.207	0.069	0.183			
	Frequency of nutrition labels reading	0.059	0.057	0.053	0.303			
**Step 3**	(Constant)	-0.300	0.572		0.600	0.016	1.094	0.365
Sociodemographic	Gender	-0.053	0.132	-0.020	0.689			
	Age range	0.079	0.062	0.065	0.203			
	Educational level	-0.098	0.074	-0.067	0.184			
Purchasing behaviour	Role in buying decisions	0.259	0.207	0.065	0.213			
	Frequency of nutrition labels reading	0.084	0.063	0.075	0.180			
Orthorexia nervosa	Eating Habits Questionnaire-21	-0.004	0.004	-0.054	0.325			
**Step 4**	(Constant)	-0.212	0.583		0.717	0.018	0.899	0.517
Sociodemographic	Gender	-0.038	0.133	-0.014	0.777			
	Age range	0.077	0.062	0.063	0.213			
	Educational level	-0.089	0.076	-0.060	0.244			
Purchasing behaviour	Role in buying decisions	0.263	0.208	0.066	0.207			
	Frequency of nutrition labels reading	0.089	0.065	0.079	0.175			
Orthorexia nervosa	Eating Habits Questionnaire-21	-0.003	0.004	-0.041	0.478			
Cognitive abilities	Need for Cognition	-0.004	0.006	-0.032	0.535			
	Subjective Ability to Evaluate Information	-0.007	0.017	-0.024	0.669			

Table S5. Hierarchical Multiple Linear Regression models explaining difference between NutrInform Battery and Nutri-Score in healthiness perception (ΔHP) of low nutritional quality yoghurt.

**ΔHP_Low nutritional quality yoghurt**	**Variables**	**Unstandardized coefficients (*B*)**	**Std.Error B**	**β**	**Sign.**	**R^2**	**F**	**Sign. Model**
**Step 1**	(Constant)	-0.253	0.314		0.422	0.006	0.829	0.478
Sociodemographic	Gender	-0.148	0.111	-0.066	0.183			
	Age range	0.025	0.052	0.024	0.632			
	Educational level	0.048	0.062	0.038	0.441			
**Step 2**	(Constant)	-0.376	0.453		0.407	0.018	1.484	0.194
Sociodemographic	Gender	-0.177	0.112	-0.079	0.116			
	Age range	0.033	0.053	0.032	0.528			
	Educational level	0.071	0.063	0.057	0.259			
Purchasing behaviour	Role in buying decisions	0.245	0.176	0.072	0.165			
	Frequency of nutrition labels reading	-0.072	0.049	-0.075	0.142			
**Step 3**	(Constant)	-0.558	0.488		0.254	0.020	1.402	0.213
Sociodemographic	Gender	-0.184	0.112	-0.082	0.102			
	Age range	0.035	0.053	0.033	0.511			
	Educational level	0.078	0.063	0.062	0.221			
Purchasing behaviour	Role in buying decisions	0.260	0.177	0.076	0.143			
	Frequency of nutrition labels reading	-0.093	0.053	-0.098	0.081			
Orthorexia nervosa	Eating Habits Questionnaire-21	0.003	0.003	0.054	0.320			
**Step 4**	(Constant)	-0.537	0.498		0.282	0.022	1.132	0.341
Sociodemographic	Gender	-0.182	0.114	-0.081	0.111			
	Age range	0.036	0.053	0.035	0.491			
	Educational level	0.088	0.065	0.070	0.175			
Purchasing behaviour	Role in buying decisions	0.251	0.178	0.073	0.160			
	Frequency of nutrition labels reading	-0.081	0.056	-0.085	0.145			
Orthorexia nervosa	Eating Habits Questionnaire-21	0.003	0.003	0.060	0.297			
Cognitive abilities	Need for Cognition	0.001	0.005	0.012	0.825			
	Subjective Ability to Evaluate Information	-0.012	0.015	-0.045	0.417			

Table S6. Hierarchical Multiple Linear Regression models explaining difference between NutrInform Battery and Nutri-Score in willingness to buy (ΔWTB) of high nutritional quality yoghurt.

**ΔWTB_High nutritional quality yoghurt**	**Variables**	**Unstandardized coefficients (*B*)**	**Std.Error B**	**β**	**Sign.**	**R^2**	**F**	**Sign. Model**
**Step 1**	(Constant)	-0.464	0.353		0.189	0.00	0.28	0.84
Sociodemographic	Gender	0.009	0.124	0.004	0.944			
	Age range	0.007	0.058	0.006	0.905			
	Educational level	0.063	0.070	0.045	0.367			
**Step 2**	(Constant)	-1.448	0.506		0.004	0.02	1.75	0.12
Sociodemographic	Gender	0.059	0.125	0.024	0.637			
	Age range	0.038	0.059	0.033	0.517			
	Educational level	0.068	0.070	0.049	0.331			
Purchasing behaviour	Role in buying decisions	**0.388**	0.197	0.101	**0.050**			
	Frequency of nutrition labels reading	**0.126**	0.055	0.118	**0.022**			
**Step 3**	(Constant)	-1.502	0.547		0.006	0.02	1.47	0.19
Sociodemographic	Gender	0.057	0.126	0.023	0.650			
	Age range	0.039	0.059	0.033	0.512			
	Educational level	0.070	0.071	0.050	0.320			
Purchasing behaviour	Role in buying decisions	**0.392**	0.198	0.102	**0.048**			
	Frequency of nutrition labels reading	**0.119**	0.060	0.112	**0.047**			
Orthorexia nervosa	Eating Habits Questionnaire-21	0.001	0.003	0.015	0.790			
**Step 4**	(Constant)	-1.519	0.558		0.007	0.02	1.10	0.36
Sociodemographic	Gender	0.054	0.127	0.022	0.671			
	Age range	0.039	0.059	0.034	0.508			
	Educational level	0.070	0.073	0.050	0.338			
Purchasing behaviour	Role in buying decisions	0.390	0.199	0.102	0.051			
	Frequency of nutrition labels reading	0.120	0.062	0.112	0.054			
Orthorexia nervosa	Eating Habits Questionnaire-21	0.001	0.004	0.012	0.832			
Cognitive abilities	Need for Cognition	0.001	0.006	0.009	0.862			
	Subjective Ability to Evaluate Information	0.000	0.016	0.000	0.996			

Table S7. Hierarchical Multiple Linear Regression models explaining difference between NutrInform Battery and Nutri-Score in willingness to buy (ΔWTB) of medium nutritional quality yoghurt.

**ΔWTB_Medium nutritional quality yoghurt**	**Variables**	**Unstandardized coefficients (*B*)**	**Std.Error B**	**β**	**Sign.**	**R^2**	**F**	**Sign. Model**
**Step 1**	(Constant)	0.358	0.385		0.352	0.008	1.063	0.365
Sociodemographic	Gender	-0.138	0.136	-0.050	0.310			
	Age range	0.002	0.063	0.002	0.975			
	Educational level	-0.109	0.076	-0.071	0.152			
**Step 2**	(Constant)	0.700	0.557		0.209	0.010	0.793	0.555
Sociodemographic	Gender	-0.156	0.138	-0.057	0.261			
	Age range	-0.009	0.065	-0.007	0.891			
	Educational level	-0.111	0.077	-0.072	0.151			
Purchasing behaviour	Role in buying decisions	-0.136	0.217	-0.032	0.533			
	Frequency of nutrition labels reading	-0.043	0.060	-0.037	0.470			
**Step 3**	(Constant)	0.949	0.601		0.115	0.013	,864	0.521
Sociodemographic	Gender	-0.146	0.138	-0.053	0.293			
	Age range	-0.011	0.065	-0.009	0.866			
	Educational level	-0.120	0.078	-0.078	0.123			
Purchasing behaviour	Role in buying decisions	-0.156	0.218	-0.037	0.475			
	Frequency of nutrition labels reading	-0.014	0.066	-0.012	0.834			
Orthorexia nervosa	Eating Habits Questionnaire-21	-0.004	0.004	-0.061	0.270			
**Step 4**	(Constant)	0.936	0.611		0.127	0.018	0.944	0.480
Sociodemographic	Gender	-0.145	0.140	-0.053	0.299			
	Age range	-0.016	0.065	-0.012	0.807			
	Educational level	-0.142	0.080	-0.092	0.075			
Purchasing behaviour	Role in buying decisions	-0.131	0.218	-0.031	0.549			
	Frequency of nutrition labels reading	-0.042	0.068	-0.036	0.540			
Orthorexia nervosa	Eating Habits Questionnaire-21	-0.004	0.004	-0.065	0.254			
Cognitive abilities	Need for Cognition	-0.005	0.006	-0.037	0.480			
	Subjective Ability to Evaluate Information	0.026	0.018	0.081	0.149			

Table S8. Hierarchical Multiple Linear Regression models explaining difference between NutrInform Battery and Nutri-Score in willingness to buy (ΔWTB) of low nutritional quality yoghurt.

**ΔWTB_Low nutritional quality yoghurt**	**Variables**	**Unstandardized coefficients (*B*)**	**Std.Error B**	**β**	**Sign.**	**R^2**	**F**	**Sign. Model**
**Step 1**	(Constant)	0.064	0.377		0.866	0.001	0.071	0.975
Sociodemographic	Gender	-0.035	0.133	-0.013	0.795			
	Age range	-0.021	0.062	-0.017	0.731			
	Educational level	-0.007	0.074	-0.004	0.929			
**Step 2**	(Constant)	0.604	0.542		0.266	0.015	1.273	0.275
Sociodemographic	Gender	-0.093	0.134	-0.035	0.490			
	Age range	-0.035	0.063	-0.028	0.584			
	Educational level	0.010	0.075	0.006	0.899			
Purchasing behaviour	Role in buying decisions	-0.044	0.211	-0.011	0.835			
	Frequency of nutrition labels reading	**-0.144**	0.058	-0.126	**0.014**			
**Step 3**	(Constant)	0.625	0.585		0.286	0.015	1.060	0.386
Sociodemographic	Gender	-0.092	0.135	-0.034	0.495			
	Age range	-0.035	0.063	-0.028	0.582			
	Educational level	0.009	0.076	0.006	0.907			
Purchasing behaviour	Role in buying decisions	-0.046	0.212	-0.011	0.830			
	Frequency of nutrition labels reading	**-0.142**	0.064	-0.124	**0.027**			
Orthorexia nervosa	Eating Habits Questionnaire-21	0.000	0.004	-0.005	0.922			
**Step 4**	(Constant)	0.840	0.594		0.158	0.025	1.286	0.249
Sociodemographic	Gender	-0.055	0.136	-0.020	0.688			
	Age range	-0.041	0.063	-0.033	0.521			
	Educational level	0.023	0.077	0.015	0.769			
Purchasing behaviour	Role in buying decisions	-0.022	0.212	-0.005	0.919			
	Frequency of nutrition labels reading	**-0.145**	0.066	-0.127	**0.030**			
Orthorexia nervosa	Eating Habits Questionnaire-21	0.002	0.004	0.025	0.663			
Cognitive abilities	Need for Cognition	-0.012	0.006	-0.098	0.060			
	Subjective Ability to Evaluate Information	-0.006	0.017	-0.018	0.749			

Table S9. Hierarchical Multiple Linear Regression models explaining difference between NutrInform Battery and Nutri-Score in healthiness perception (ΔHP) of high nutritional quality jam.

**ΔHP_High nutritional quality jam**	**Variables**	**Unstandardized coefficients (*B*)**	**Std.Error B**	**β**	**Sign.**	**R^2**	**F**	**Sign. Model**
**Step 1**	(Constant)	0.020	0.404		0.960	**0.024**	3.002	**0.031**
Sociodemographic	Gender	-0.020	0.130	-0.008	0.876			
	Age range	**0.159**	0.061	0.135	**0.010**			
	Educational level	-0.094	0.075	-0.065	0.212			
**Step 2**	(Constant)	0.215	0.555		0.698	0.031	1.302	0.273
Sociodemographic	Gender	-0.035	0.131	-0.014	0.790			
	Age range	**0.152**	0.062	0.129	**0.014**			
	Educational level	-0.115	0.076	-0.080	0.131			
Purchasing behaviour	Role in buying decisions	-0.227	0.210	-0.058	0.281			
	Frequency of nutrition labels reading	0.059	0.058	0.053	0.311			
**Step 3**	(Constant)	0.370	0.606		0.542	0.032	0.406	0.525
Sociodemographic	Gender	-0.036	0.131	-0.014	0.784			
	Age range	**0.152**	0.062	0.129	**0.014**			
	Educational level	-0.116	0.076	-0.080	0.129			
Purchasing behaviour	Role in buying decisions	-0.226	0.210	-0.058	0.283			
	Frequency of nutrition labels reading	0.058	0.058	0.053	0.316			
Orthorexia nervosa	Eating Habits Questionnaire-21	-0.002	0.003	-0.033	0.525			
**Step 4**	(Constant)	0.719	0.648		0.268	0.039	1.257	0.286
Sociodemographic	Gender	-0.019	0.132	-0.008	0.884			
	Age range	**0.149**	0.062	0.126	**0.016**			
	Educational level	-0.126	0.076	-0.087	0.101			
Purchasing behaviour	Role in buying decisions	-0.260	0.211	-0.066	0.218			
	Frequency of nutrition labels reading	0.060	0.058	0.054	0.299			
Orthorexia nervosa	Eating Habits Questionnaire-21	0.000	0.004	0.001	0.986			
Cognitive abilities	Need for Cognition	-0.004	0.007	-0.031	0.570			
	Subjective Ability to Evaluate Information	-0.024	0.017	-0.078	0.161			

Table S10. Hierarchical Multiple Linear Regression models explaining difference between NutrInform Battery and Nutri-Score in healthiness perception (ΔHP) of medium nutritional quality jam.

**ΔHP_Medium nutritional quality jam**	**Variables**	**Unstandardized coefficients (*B*)**	**Std.Error B**	**β**	**Sign.**	**R^2**	**F**	**Sign. Model**
**Step 1**	(Constant)	0.179	0.380		0.639	0.000	0.039	0.990
Sociodemographic	Gender	0.002	0.122	0.001	0.986			
	Age range	0.004	0.058	0.004	0.943			
	Educational level	0.024	0.070	0.018	0.733			
**Step 2**	(Constant)	-0.372	0.523		0.477	0.008	0.561	0.730
Sociodemographic	Gender	0.024	0.123	0.010	0.848			
	Age range	0.014	0.058	0.013	0.810			
	Educational level	0.043	0.072	0.032	0.547			
Purchasing behaviour	Role in buying decisions	0.324	0.198	0.089	0.102			
	Frequency of nutrition labels reading	0.019	0.055	0.018	0.735			
**Step 3**	(Constant)	-0.337	0.572		0.556	0.008	0.470	0.830
Sociodemographic	Gender	0.023	0.123	0.010	0.850			
	Age range	0.014	0.058	0.013	0.811			
	Educational level	0.043	0.072	0.032	0.548			
Purchasing behaviour	Role in buying decisions	0.324	0.198	0.089	0.103			
	Frequency of nutrition labels reading	0.018	0.055	0.018	0.737			
Orthorexia nervosa	Eating Habits Questionnaire-21	0.000	0.003	-0.008	0.877			
**Step 4**	(Constant)	-0.490	0.612		0.424	0.011	0.507	0.851
Sociodemographic	Gender	0.025	0.125	0.011	0.843			
	Age range	0.014	0.058	0.013	0.814			
	Educational level	0.047	0.072	0.035	0.517			
Purchasing behaviour	Role in buying decisions	0.342	0.199	0.094	0.087			
	Frequency of nutrition labels reading	0.018	0.055	0.018	0.739			
Orthorexia nervosa	Eating Habits Questionnaire-21	-0.002	0.003	-0.025	0.661			
Cognitive abilities	Need for Cognition	-0.002	0.006	-0.014	0.807			
	Subjective Ability to Evaluate Information	0.018	0.016	0.062	0.269			

Table S11. Hierarchical Multiple Linear Regression models explaining difference between NutrInform Battery and Nutri-Score in healthiness perception (ΔHP) of low nutritional quality jam.

**ΔHP_Low nutritional quality jam**	**Variables**	**Unstandardized coefficients (*B*)**	**Std.Error B**	**β**	**Sign.**	**R^2**	**F**	**Sign. Model**
**Step 1**	(Constant)	0.117	0.455		0.796	0.004	0.544	0.652
Sociodemographic	Gender	0.137	0.146	0.049	0.348			
	Age range	-0.016	0.069	-0.012	0.821			
	Educational level	0.074	0.084	0.046	0.381			
**Step 2**	(Constant)	-0.082	0.626		0.896	0.009	0.672	0.645
Sociodemographic	Gender	0.151	0.147	0.054	0.305			
	Age range	-0.009	0.070	-0.007	0.899			
	Educational level	0.094	0.086	0.059	0.273			
Purchasing behaviour	Role in buying decisions	0.217	0.237	0.050	0.360			
	Frequency of nutrition labels reading	-0.052	0.066	-0.042	0.431			
**Step 3**	(Constant)	-0.037	0.684		0.957	0.009	0.563	0.760
Sociodemographic	Gender	0.151	0.147	0.054	0.306			
	Age range	-0.009	0.070	-0.007	0.899			
	Educational level	0.094	0.086	0.059	0.274			
Purchasing behaviour	Role in buying decisions	0.217	0.237	0.050	0.360			
	Frequency of nutrition labels reading	-0.052	0.066	-0.042	0.430			
Orthorexia nervosa	Eating Habits Questionnaire-21	-0.001	0.004	-0.009	0.868			
**Step 4**	(Constant)	-0.170	0.734		0.816	0.010	0.470	0.877
Sociodemographic	Gender	0.136	0.150	0.049	0.362			
	Age range	-0.007	0.070	-0.005	0.925			
	Educational level	0.098	0.087	0.061	0.256			
Purchasing behaviour	Role in buying decisions	0.227	0.239	0.052	0.342			
	Frequency of nutrition labels reading	-0.053	0.066	-0.043	0.418			
Orthorexia nervosa	Eating Habits Questionnaire-21	-0.001	0.004	-0.019	0.732			
Cognitive abilities	Need for Cognition	0.004	0.007	0.033	0.549			
	Subjective Ability to Evaluate Information	0.002	0.019	0.006	0.914			

Table S12. Hierarchical Multiple Linear Regression models explaining difference between NutrInform Battery and Nutri-Score in willingness to buy (ΔWTB) of high nutritional quality jam.

**ΔWTB_High nutritional quality jam**	**Variables**	**Unstandardized coefficients (*B*)**	**Std.Error B**	**β**	**Sign.**	**R^2**	**F**	**Sign. Model**
**Step 1**	(Constant)	-0.371	0.402		0.357	0.032	4.088	0.007
Sociodemographic	Gender	0.187	0.129	0.074	0.150			
	Age range	**0.164**	0.061	0.139	**0.008**			
	Educational level	-0.087	0.074	-0.060	0.243			
**Step 2**	(Constant)	0.436	0.551		0.429	0.046	3.489	0.004
Sociodemographic	Gender	0.156	0.130	0.062	0.231			
	Age range	**0.150**	0.061	0.127	**0.015**			
	Educational level	-0.114	0.076	-0.079	0.131			
Purchasing behaviour	Role in buying decisions	**-0.468**	0.208	-0.119	**0.025**			
	Frequency of nutrition labels reading	-0.031	0.058	-0.028	0.593			
**Step 3**	(Constant)	0.521	0.602		0.387	0.046	2.921	0.009
Sociodemographic	Gender	0.155	0.130	0.062	0.233			
	Age range	**0.150**	0.061	0.127	**0.015**			
	Educational level	-0.115	0.076	-0.080	0.130			
Purchasing behaviour	Role in buying decisions	**-0.467**	0.208	-0.119	**0.026**			
	Frequency of nutrition labels reading	-0.031	0.058	-0.028	0.589			
Orthorexia nervosa	Eating Habits Questionnaire-21	-0.001	0.003	-0.018	0.726			
**Step 4**	(Constant)	0.782	0.644		0.225	0.051	2.436	0.014
Sociodemographic	Gender	0.161	0.131	0.064	0.222			
	Age range	**0.149**	0.061	0.126	**0.016**			
	Educational level	-0.122	0.076	-0.084	0.110			
Purchasing behaviour	Role in buying decisions	**-0.495**	0.210	-0.126	**0.019**			
	Frequency of nutrition labels reading	-0.030	0.058	-0.027	0.601			
Orthorexia nervosa	Eating Habits Questionnaire-21	0.001	0.004	0.008	0.886			
Cognitive abilities	Need for Cognition	0.000	0.007	-0.004	0.947			
	Subjective Ability to Evaluate Information	-0.023	0.017	-0.076	0.168			

Table S13. Hierarchical Multiple Linear Regression models explaining difference between NutrInform Battery and Nutri-Score in willingness to buy (ΔWTB) of medium nutritional quality jam.

**ΔWTB_Medium nutritional quality jam**	**Variables**	**Unstandardized coefficients (*B*)**	**Std.Error B**	**β**	**Sign.**	**R^2**	**F**	**Sign. Model**
**Step 1**	(Constant)	0.774	0.370		0.037	0.006	0.795	0.498
Sociodemographic	Gender	-0.011	0.119	-0.005	0.927			
	Age range	-0.054	0.056	-0.051	0.334			
	Educational level	-0.088	0.069	-0.067	0.198			
**Step 2**	(Constant)	0.416	0.510		0.415	0.009	0.684	0.636
Sociodemographic	Gender	0.001	0.120	0.000	0.993			
	Age range	-0.049	0.057	-0.046	0.387			
	Educational level	-0.080	0.070	-0.061	0.252			
Purchasing behaviour	Role in buying decisions	0.178	0.193	0.050	0.358			
	Frequency of nutrition labels reading	0.031	0.053	0.031	0.563			
**Step 3**	(Constant)	0.304	0.558		0.585	0.010	0.610	0.722
Sociodemographic	Gender	0.002	0.120	0.001	0.988			
	Age range	-0.049	0.057	-0.046	0.387			
	Educational level	-0.080	0.070	-0.061	0.254			
Purchasing behaviour	Role in buying decisions	0.177	0.193	0.050	0.360			
	Frequency of nutrition labels reading	0.031	0.053	0.031	0.558			
Orthorexia nervosa	Eating Habits Questionnaire-21	0.002	0.003	0.026	0.617			
**Step 4**	(Constant)	-0.007	0.596		0.991	0.016	0.734	0.661
Sociodemographic	Gender	-0.016	0.122	-0.007	0.897			
	Age range	-0.046	0.057	-0.043	0.415			
	Educational level	-0.071	0.070	-0.054	0.313			
Purchasing behaviour	Role in buying decisions	0.207	0.194	0.058	0.288			
	Frequency of nutrition labels reading	0.029	0.053	0.029	0.585			
Orthorexia nervosa	Eating Habits Questionnaire-21	0.000	0.003	-0.007	0.906			
Cognitive abilities	Need for Cognition	0.004	0.006	0.040	0.471			
	Subjective Ability to Evaluate Information	0.019	0.015	0.068	0.228			

Table S14. Hierarchical Multiple Linear Regression models explaining difference between NutrInform Battery and Nutri-Score in willingness to buy (ΔWTB) of low nutritional quality jam.

**ΔWTB_Low nutritional quality jam**	**Variables**	**Unstandardized coefficients (*B*)**	**Std.Error B**	**β**	**Sign.**	**R^2**	**F**	**Sign. Model**
**Step 1**	(Constant)	0.326	0.441		0.460	0.006	0.798	0.495
Sociodemographic	Gender	0.145	0.142	0.054	0.306			
	Age range	-0.074	0.067	-0.058	0.273			
	Educational level	0.040	0.082	0.025	0.627			
**Step 2**	(Constant)	0.184	0.608		0.763	0.007	0.504	0.773
Sociodemographic	Gender	0.149	0.143	0.055	0.297			
	Age range	-0.072	0.068	-0.056	0.288			
	Educational level	0.041	0.083	0.027	0.620			
Purchasing behaviour	Role in buying decisions	0.058	0.230	0.014	0.800			
	Frequency of nutrition labels reading	0.019	0.064	0.016	0.760			
**Step 3**	(Constant)	0.170	0.664		0.798	0.007	0.419	0.866
Sociodemographic	Gender	0.149	0.143	0.055	0.298			
	Age range	-0.072	0.068	-0.056	0.288			
	Educational level	0.041	0.083	0.027	0.621			
Purchasing behaviour	Role in buying decisions	0.058	0.230	0.014	0.800			
	Frequency of nutrition labels reading	0.020	0.064	0.016	0.759			
Orthorexia nervosa	Eating Habits Questionnaire-21	0.000	0.004	0.003	0.959			
**Step 4**	(Constant)	0.260	0.712		0.715	0.009	0.412	0.913
Sociodemographic	Gender	0.168	0.145	0.062	0.248			
	Age range	-0.075	0.068	-0.059	0.271			
	Educational level	0.038	0.084	0.024	0.653			
Purchasing behaviour	Role in buying decisions	0.055	0.232	0.013	0.812			
	Frequency of nutrition labels reading	0.021	0.064	0.018	0.738			
Orthorexia nervosa	Eating Habits Questionnaire-21	0.001	0.004	0.009	0.869			
Cognitive abilities	Need for Cognition	-0.006	0.007	-0.048	0.390			
	Subjective Ability to Evaluate Information	0.006	0.018	0.018	0.748			
